# Population and single‐cell analyses reveal immune cell‐specific expression profiles associated with Alzheimer's disease risk

**DOI:** 10.1002/alz.71282

**Published:** 2026-03-22

**Authors:** Joni V. Lindbohm, Martin Stražar, Hang‐Mao Lee, Orr Ashenberg, Nina Mars, Pyry N. Sipilä, Samuli Ripatti, Dan Graham, Mika Kivimäki, Ramnik J. Xavier

**Affiliations:** ^1^ Clinicum, Department of Public Health University of Helsinki Helsinki Finland; ^2^ Broad Institute of the Massachusetts Institute of Technology and Harvard University Cambridge Massachusetts USA; ^3^ UCL Brain Sciences University College London London UK; ^4^ Institute for Molecular Medicine Finland (FIMM), HiLIFE University of Helsinki Helsinki Finland; ^5^ Center for Computational and Integrative Biology Massachusetts General Hospital and Harvard Medical School Boston Massachusetts USA; ^6^ Department of Molecular Biology Massachusetts General Hospital and Harvard Medical School Massachusetts USA

**Keywords:** Alzheimer's disease, brain infiltrating, colocalization, immune system, Mendelian randomization, peripheral blood immune cells, single cell, spatial transcriptomics

## Abstract

**INTRODUCTION:**

Dysregulation of the peripheral immune system may increase Alzheimer's disease (AD) risk, but the underlying cell type‐specific mechanisms remain unclear.

**METHODS:**

We conducted Mendelian randomization and colocalization analyses of 4489 genes using single‐cell expression quantitative trait locus data from unstimulated and stimulated peripheral immune cells, integrated with an AD genome‐wide association study (*N* = 455,258). Spatial transcriptomics of brain tissue samples was used to identify brain‐infiltrating immune cells.

**RESULTS:**

Thirteen genes were associated with AD risk. Expression of *BIN1*, *CTSW*, *CTSH*, *HLA‐DRB1*, *TSTD1*, *PLEKHA1*, and *SCIMP* increased AD risk, while *EPHA1‐AS1*, *FCER1G*, *FIBP*, *KAT8*, *STX4*, and *HLA‐DQA1* reduced it. These associations were peripheral immune cell type and state specific. *PLEKHA1* and *TSTD1* were upregulated and *FIBP* downregulated in natural killer and T cells in AD brain tissue.

**DISCUSSION:**

These findings link immune cell‐specific gene expression to AD risk across activation states and within brain‐infiltrating immune cells, highlighting potential targets for immune‐based AD prevention and treatment.

## Highlights

- Thirteen peripheral immune cell gene expressions were linked to Alzheimer's disease (AD) risk.
- Ten were linked to AD before and nine after immune activation.
- Three of 13 genes had higher expression in brain‐infiltrating NK and T cells in AD.
- Of those 3, *PLEKHA1* and *TSTD1* increased and *FIBP* decreased AD risk.
- The three genes are potential AD therapeutic targets in preclinical and clinical stages.

## 1 BACKGROUND

Alzheimer's disease (AD) is a neurodegenerative disorder characterized by the accumulation of amyloid beta plaques, tau tangles, and chronic neuroinflammation that develop during a decades‐long asymptomatic phase before the onset of cognitive symptoms.^1^ As therapies targeting amyloid and tau have shown only modest disease‐modifying effects,^2^ research has increasingly focused on alternative drug targets.

One hypothesis suggests that dysfunction of the peripheral immune system may contribute to AD pathogenesis.^3^ Consistent with this, genome‐wide association studies (GWASs) have identified many immune‐related genes among the top genetic associations with AD.^4^ Additionally, studies using plasma proteomics and Mendelian randomization have linked immune‐related circulating proteins to both near‐ and long‐term risks of dementias, including AD.^5^, ^6^, ^7^ Emulated randomized controlled trials have further suggested that peripherally acting anti‐inflammatory drugs like methotrexate and tumor necrosis factor alpha inhibitors could reduce the risk of AD.^6^ Furthermore, individuals with severe infections requiring hospital treatment have been shown to have increased risk of AD and other dementias, even when the infection occurred more than 20 years before dementia onset.^8^, ^9^

To date, however, cellular‐level evidence linking dysregulation of the peripheral immune system with neuroinflammation and AD remains limited. Some studies have suggested that increased antigen presentation and elevated levels of pro‐inflammatory memory CD4^+^ and CD8^+^ T cells in blood and cerebrospinal fluid^3^ may be associated with hippocampal damage and cognitive symptoms in AD patients.^5^, ^10^ To our knowledge, however, no studies have examined whether immune cell‐specific expression profiles, before and after stimulation, in peripheral blood and brain‐infiltrating immune cells are related to AD risk.

To identify blood immune cell RNA expression profiles that predispose to AD across different domains of immune activation, we performed Mendelian randomization (MR) and colocalization analyses using unstimulated and stimulated blood single‐cell RNA (scRNA) data and data from AD GWASs. We also analyzed the expression of the identified risk genes in putatively brain‐infiltrating immune cells using spatial transcriptomics of *post mortem* brain tissue. Our findings identify 13 peripheral and three brain immune cell‐ and stimulus‐specific genes associated with increased AD risk across early and late disease phases.

## 2 METHODS

### 2.1 Mendelian randomization

In MR, exposure instruments were expression quantitative trait loci (eQTLs) for scRNA expression levels of unstimulated and stimulated peripheral blood mononuclear cells (PBMCs) and were extracted from three different studies. Unstimulated PBMC data were extracted from the OneK1K study that consisted of 982 healthy individuals of Northern European ancestry and 1,267,758 cells.^11^ CD4^+^ T cells stimulated with anti‐CD3/anti‐CD28 human T‐Activator Dynabeads (Invitrogen) were extracted from data by Soskic et al.^12^ and included 655,349 CD4^+^ T cells from 119 healthy individuals of British ancestry. RNA expression for these cells was measured after 16 h, 40 h, and 5 days of stimulation. Pathogen‐stimulated PBMC data were extracted from the Lifelines study by Oelen et al.^13^ and included 1.3 million cells from 120 individuals of the Northern Netherlands population cohort Lifelines. The eQTLs were determined after 3 and 24 h of in vitro stimulation with *C. albicans*, *M. tuberculosis*, or *P. aeruginosa*. AD GWASs with 71,880 cases and 383,378 controls of European ancestry by Jansen et al.^14^ provided instruments for AD outcomes in MR analyses.

Two‐sample MR was used to analyze associations between biomarkers and outcomes.^15^ The analyses estimated main effects using Wald ratio or inverse variance‐weighted analyses^16^ and used weighted median, weighted mode, and MR Egger as sensitivity analyses when number of single‐nucleotide polymorphisms (SNPs) exceeded 2.^15^ We applied a false discovery rate (FDR) correction of 5% for the total number of tests conducted within each PBMC study in MR. The analyses were done using TwoSampleMR and MRInstruments R packages. In all analyses, we used individuals of European ancestry, a clumping cut‐off *R* ^2^ of 0.001, and a 500‐kb window for unstimulated and 10‐kb window for more limited stimulated data. Linkage disequilibrium (LD) proxies were searched with an *R* ^2^ = 0.6 threshold and a proxy split size of 500. The biomarkers and outcome alleles were harmonized by inferring from positive strand alleles using allele frequencies for palindromes. For these analyses, we used the R statistical software (versions 4.3.1 and 4.1.0). The novelty of MR findings was examined by a systematic PubMed search using the following search terms: (dementia OR Alzheim*) AND (Entrez gene symbol) without limitations.

### 2.2 Pathway enrichment analyses

To identify biological pathways enriched for the FDR significant genes from our MR analyses, we used the clusterprofiler R package and hypergeometric test.^17^ These analyses were done separately for each cell and stimulation type, and the enrichment was studied for all cell‐specific genes that passed FDR correction in MR analyses while the background gene set was all the genes analyzed. Enriched pathways were searched from all main Gene Ontology (GO) databases including biological processes, cellular components, and molecular function. Only pathways that passed a cut‐off FDR *p* value of <0.05 were considered.

### 2.3 Colocalization analyses

Colocalization analyses were performed to validate the MR results and were done with the coloc package in R. Summary statistics for the PBMC scRNA and AD GWASs were analyzed to identify overlapping SNPs. Because only few SNPs were available for the genes studied, we computed the posterior probability for a single shared variant (H4) and used a probability of 0.7 as colocalization cut‐off for causal variants.

**RESEARCH IN CONTEXT**

1. **Systematic review**: We searched PubMed for Mendelian randomization studies on single peripheral blood immune cells and AD up to January 8, 2025. We found no studies that examined immune cell‐specific expression profiles in AD before and after immune stimulation or any that evaluated such expression in brain‐infiltrating peripheral immune cells.
2. **Interpretation**: We identified 13 genes, of which 10 were linked to AD before and nine after immune system stimulus. Of the 13 risk genes, *FIBP*, *PLEKHA1*, and *TSTD1* were associated with AD risk in both unstimulated and stimulated natural killer, CD4^+^ T, and CD8^+^ T cells and were differentially expressed in the same cell types infiltrating the brain.
3. **Future directions**: Future experimental work is needed to clarify how these genes function in immune cells and to determine whether the 13 genes or their protein products are viable targets for AD prevention or treatment.

### 2.4 Spatial brain single cell analyses

#### 2.4.1 Deconvolution and cell‐type assignment

We used spatial transcriptomic (ST) data from the brain tissue dataset of van Olst et al., which included 123,237 spots from 19 AD patients and six healthy controls.^18^ For our analyses, we included only those six AD patients who had not received AN1792 treatment and the six healthy controls, yielding a final dataset of 58,416 spots (Table S1). Spot‐level cell‐type composition was examined using Cell2location^19^ (c2l). Reference scRNA sequencing data for PBMCs were obtained from the Human Immune Health Atlas dataset^20^ and for brain cells from the Human M1 10x dataset.^21^ The datasets underwent standard quality control (removal of low‐quality cells, including low‐complexity libraries and extreme mitochondrial content) and were then randomly down‐sampled to a maximum of 5000 cells per file. The processed brain and immune datasets were concatenated to form a joint reference used for cell‐type deconvolution. A reference regression model was trained on merged single‐cell data from human brain and PBMCs, using the following gene‐filtering parameters: cell_count_cutoff = 5, cell_percentage_cutoff2 = 0.03, and nonz_mean_cutoff = 1.12. Target cell types were defined using the cell_type_alias_label from the Human M1 10x dataset and the AIFI_L1 classification from the Human Immune Health Atlas.

The reference model was trained for 10 epochs corresponding to the elbow point of the loss curve. Each ST sample was subsequently deconvoluted with *N*_cells_per_location = 7, detection_alpha = 20 for 30,000 epochs, and posterior mean gene‐expression signatures (means_per_cluster_mu_fg) derived from the reference model. Lower‐bound abundance estimates (q05cell_abundance_w_sf_means_per_cluster_mu_fg_*) were used to derive cell‐type proportions per sample. Cell subtypes were aggregated by summing spot abundance into major classes – astrocyte, B cell, dendritic cell, double negative T cell, endothelial cell, erythrocyte, excitatory neuron, inhibitory neuron, innate lymphocytic cell, microglia, monocyte, natural killer (NK) cell, oligodendrocyte, oligodendrocytic precursor cell, platelet, progenitor cell, CD4^+^ T cell, CD8^+^ T cell, T‐regulatory cells, and vascular leptomeningeal cells – and then converted to percentages. Sample‐specific cell‐type proportions were used as percentile thresholds to identify spots positive for each major cell type in each sample.

#### 2.4.2 Estimation of immune cell percentages in tissue slices

For each sample and immune cell type, Visium spots were classified as immune‐cell‐positive based on co‐assignment with the respective immune cell type and were further stratified by the presence or absence of an endothelial cell signature. For each combination of sample, immune cell type, and endothelial status, we calculated the percentage of immune‐positive spots by dividing the number of immune‐positive spots by the total number of retained spots in that sample. Retained spots were defined as Visium spots located within the tissue mask and excluding spots on the outermost array rows or columns. For each immune cell type and endothelial status, differences between AD and control samples were assessed using a pooled two‐proportion *z*‐test, and two‐sided *p* values are reported.

#### 2.4.3 Spatial overlay of marker expression

We used Scanpy to calculate gene module scores of immune cells and overlaid them on co‐registered hematoxylin and eosin images. Marker panels included CD4^+^ T cell (CD3D, CD3E, CD2, TRAC, CD4, IL7R, CCR7, TCF7); CD8^+^ T cell (CD3D, CD3E, CD2, TRAC, CD8A, CD8B, GZMA, GZMB, PRF1); and NK cell (NKG7, GNLY, PRF1, GZMB, KLRD1). Raw counts were normalized and log‐transformed, and scanpy.tl.score_genes were used to compute background‐adjusted panel scores.

#### 2.4.4 Differential expression analyzed with model‐based analysis of single‐cell transcriptomics (MAST)

We compared AD and healthy controls separately for each target cell type (major types and T‐cell subtypes). For a given type and for each sample, all spots assigned to the type were collected, excluding (i) endothelial‐positive spots and (ii) spots located on the outermost perimeter of the capture grid. Count matrices were subset to the remaining spots and concatenated across samples. We retained genes if detected in ≥20 spots across all test and reference samples. To ensure sufficient replication, we required ≥10 spots contributed by each group and ≥50 spots in total after subsetting; cell types failing these criteria were not modeled.

We transformed counts to log_2_ counts per million (log_2_CPM) using edgeR with a prior count of 1. For each spot, a cellular detection rate (CDR) covariate was computed as the scaled log1p of the number of detected genes and included as a nuisance regressor.

For each eligible cell type, we constructed a MAST SingleCellAssay object comprising the log_2_CPM expression matrix and spot‐level metadata (sample, condition, CDR). A two‐part generalized linear model was fitted for each gene, modeling (i) the probability of expression detection (logistic component) and (ii) the expression level conditional on detection (Gaussian component), with condition and CDR as predictors. If this model failed to converge, we used a fallback specification (∼ condition + CDR + sample), adding a sample term as an additional fixed effect. Differential expression was assessed via a likelihood‐ratio test (LRT) on the condition coefficient, testing whether the estimated effect of the numerator group (AD) relative to the healthy reference group differed significantly from zero.

The resulting hurdle and continuous component *p* values were combined by taking their minimum and adjusted using the Benjamini–Hochberg procedure to obtain *q* values. We then computed an average log_2_ fold change for each gene as the difference between the mean log_2_CPM values in the AD and healthy reference groups.

### 2.5 Open Targets analyses

Medications that change the levels of the proteins coded by 13 risk genes were searched from the Open Targets database (https://www.opentargets.org/) using UniProt protein and Entrez gene symbols.

## 3 RESULTS

**FIGURE 1:**
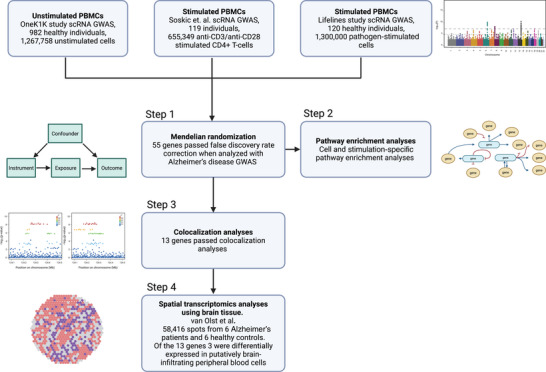
Flowchart of study design. The study aimed to identify blood immune cell profiles that predisposed to Alzheimer's disease (AD) across different domains of immune activation. Step 1: Expression quantitative trait loci (eQTL) data from genome‐wide association studies (GWASs) of unstimulated and stimulated blood immune cells and AD GWAS data were used for Mendelian randomization (MR) analyses to identify immune cell‐ and stimulus‐specific genes associated with increased AD risk. Step 2: Pathway enrichment analyses were used to summarize the biological processes represented by genes that met false discovery rate (FDR) correction criteria in MR analyses. Step 3: Colocalization analyses linked the MR‐significant findings to AD risk loci, providing evidence for shared causal variants. Step 4: Spatial transcriptomics of brain tissue samples was used to examine whether the MR‐ and colocalization‐significant genes were also expressed in putatively brain‐infiltrating blood cells, providing insight into whether the causal genes were likely to act within the peripheral or central immune system.

Figure 1 illustrates the overall study design, and Figures 2 and 3 present the results from cell‐specific MR analyses (detailed results in Figures S1–S3). Among the 2909 unstimulated PBMC genes with available cis‐eQTLs, 10 passed the FDR threshold (*p* < 0.0007) and met the colocalization probability criterion (H4 > 0.7) with AD risk variants. Of the 936 eQTLs identified from CD4^+^ T cells stimulated in vitro with a T‐cell activator, six satisfied the FDR threshold (*p* < 0.0004) and the colocalization cut‐off (H4 > 0.7) with AD risk variants. Similarly, among the 644 eQTLs derived from pathogen‐stimulated cells, four passed the FDR threshold (*p* < 0.0006) and colocalized with AD risk variants (H4 > 0.7). After removing duplicates, the stimulated and unstimulated datasets yielded 13 unique genes that met both the MR FDR threshold and the colocalization criterion: *BIN1*, *CTSW*, *TSTD1*, *PLEKHA1*, *SCIMP*, *FIBP*, *KAT8*, *STX4*, *HLA‐DQA1 CTSH*, *HLA‐DRB1*, *FCER1G* and *EPHA1‐AS1*.

FIGURE 2Mendelian randomization analyses examining Alzheimer's disease (AD) risk associated with genes expressed in unstimulated CD4^+^ T cells and stimulated CD4^+^ T cells (anti‐CD3/anti‐CD28 human T‐cell activator or pathogens) across different time windows. (A) Schematic illustration of genes whose increased RNA expression is associated with higher AD risk (in red, marked with +) and genes whose expression is associated with lower risk (in blue, marked with −). (B) Corresponding odds ratios for dementia risk for each cell type across stimulation time windows. All genes displayed passed both false discovery rate–corrected Mendelian randomization analyses (FDR < 0.05) and colocalization analysis (posterior probability for H4 > 0.7).

**Figure:**
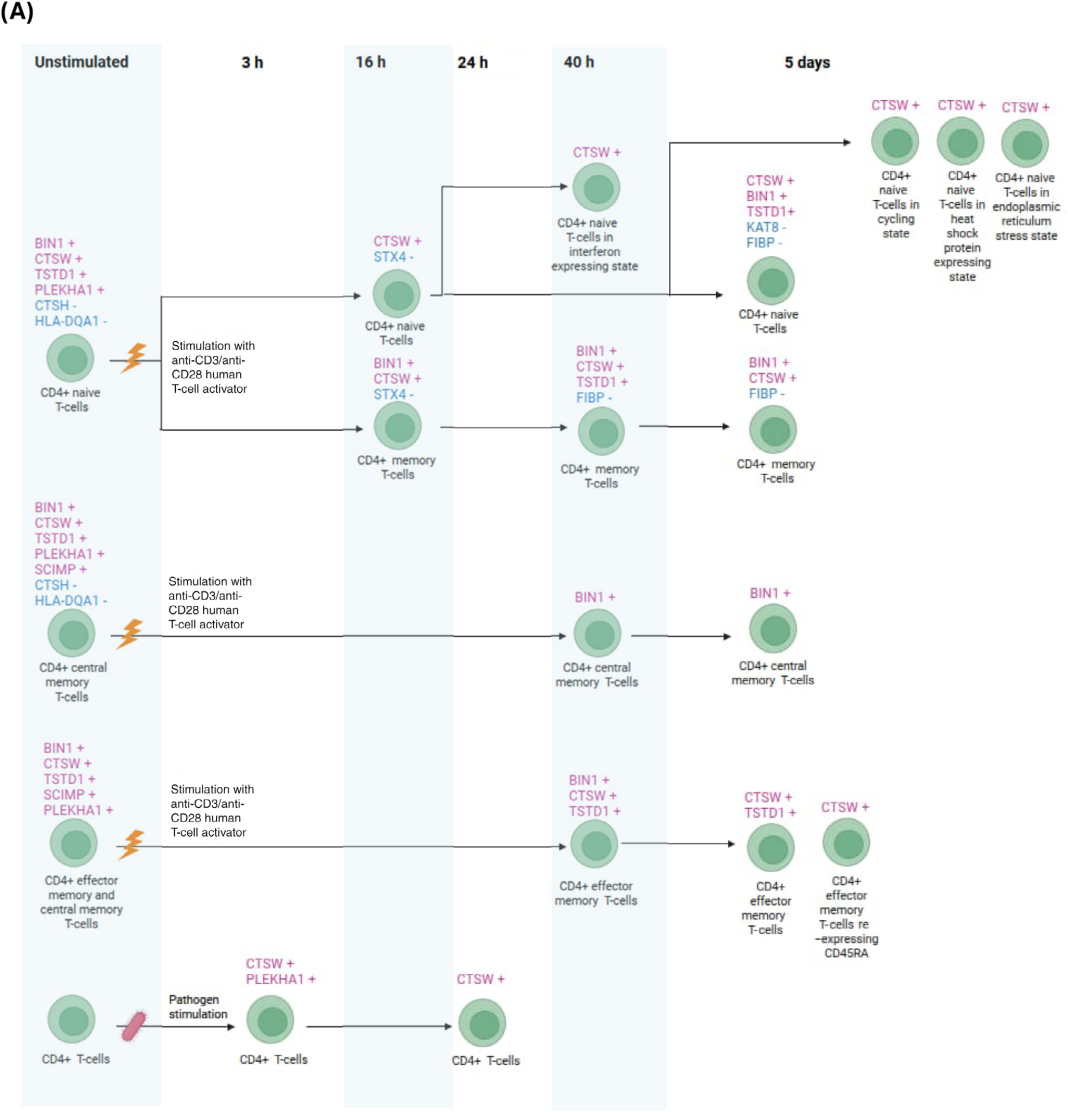

**Figure:**
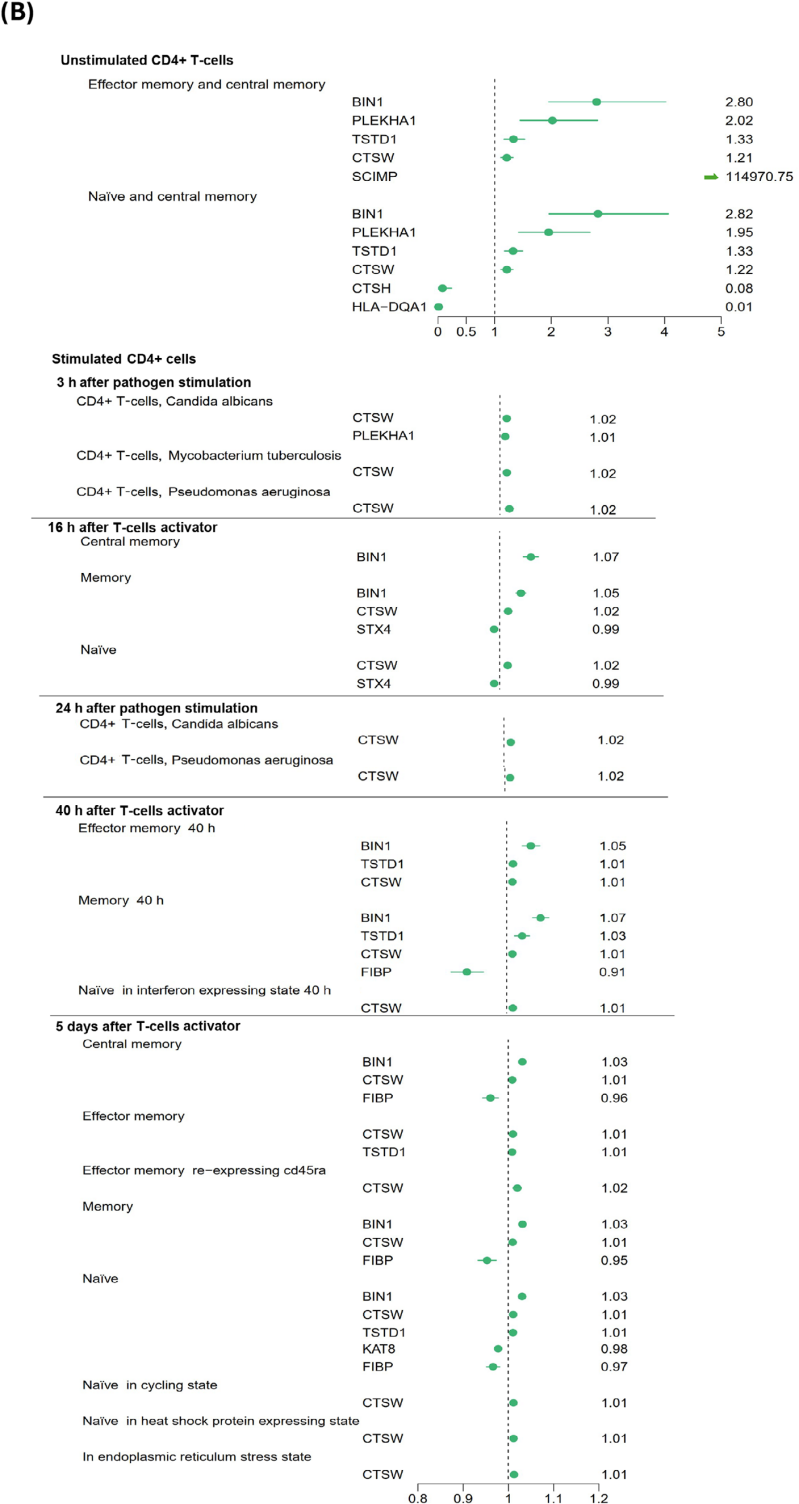

FIGURE 3Mendelian randomization analyses examining Alzheimer's disease (AD) risk associated with genes expressed in unstimulated and pathogen‐stimulated CD8^+^ T cells, B cells, natural killer cells, monocytes, and dendritic cells across different time windows. (A) Schematic illustration of genes whose increased RNA expression is associated with higher AD risk (in red, marked with +) and genes whose expression is associated with lower risk (in blue, marked with −). (B) Corresponding odds ratios for dementia for each cell type across stimulation time windows. All genes displayed passed both false discovery rate–corrected Mendelian randomization analyses (FDR < 0.05) and colocalization analysis (posterior probability for H4 > 0.7).

**Figure:**
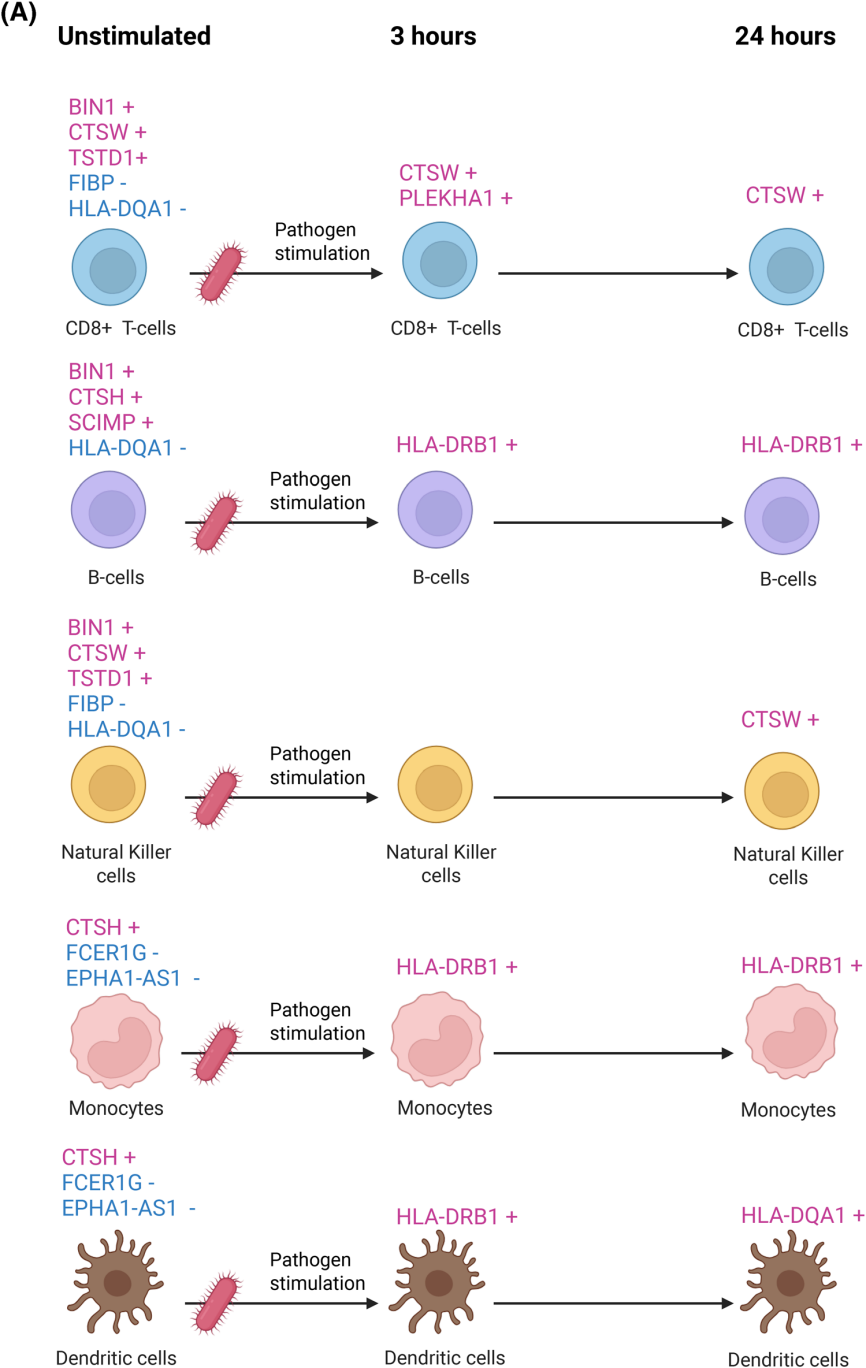

**Figure:**
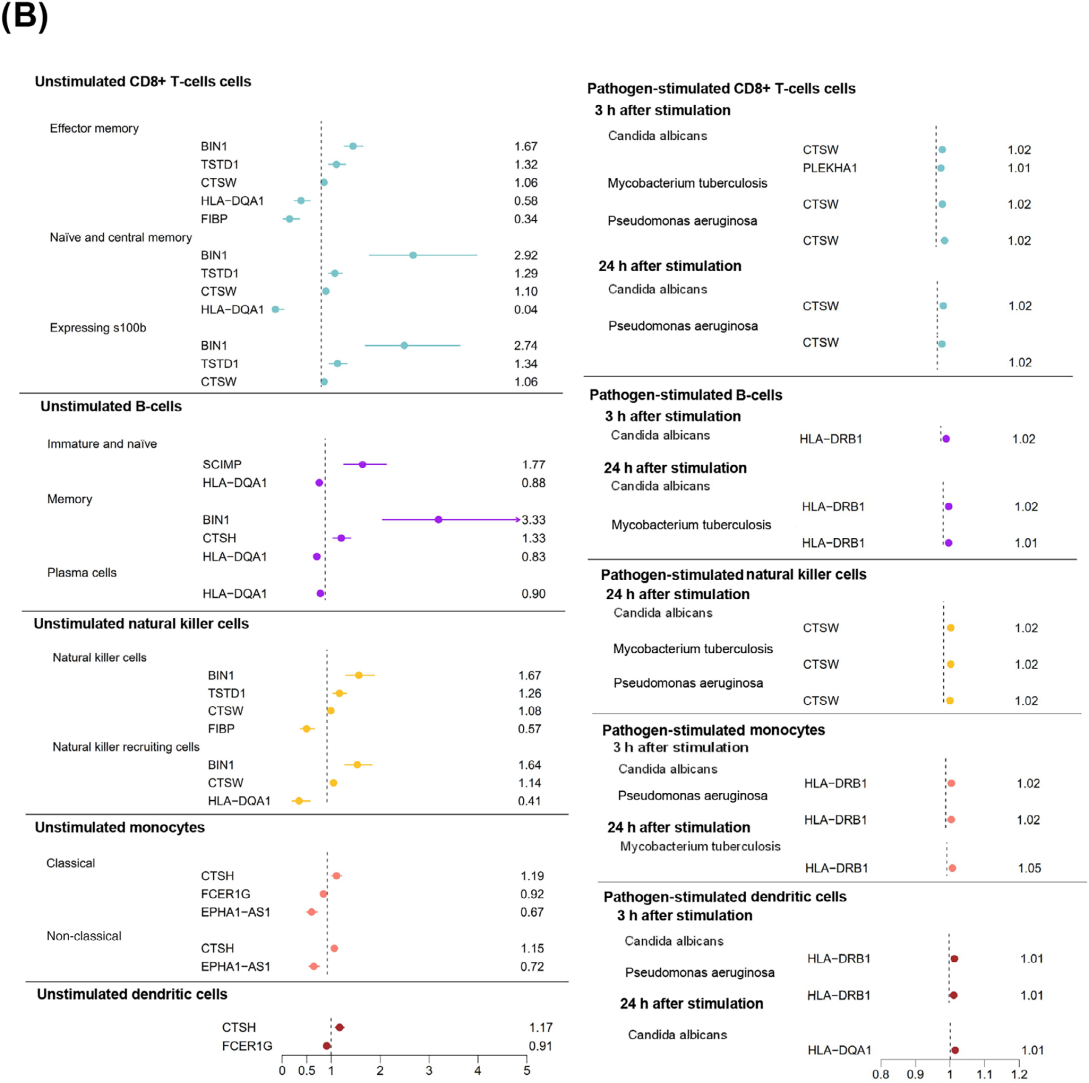

**FIGURE 4:**
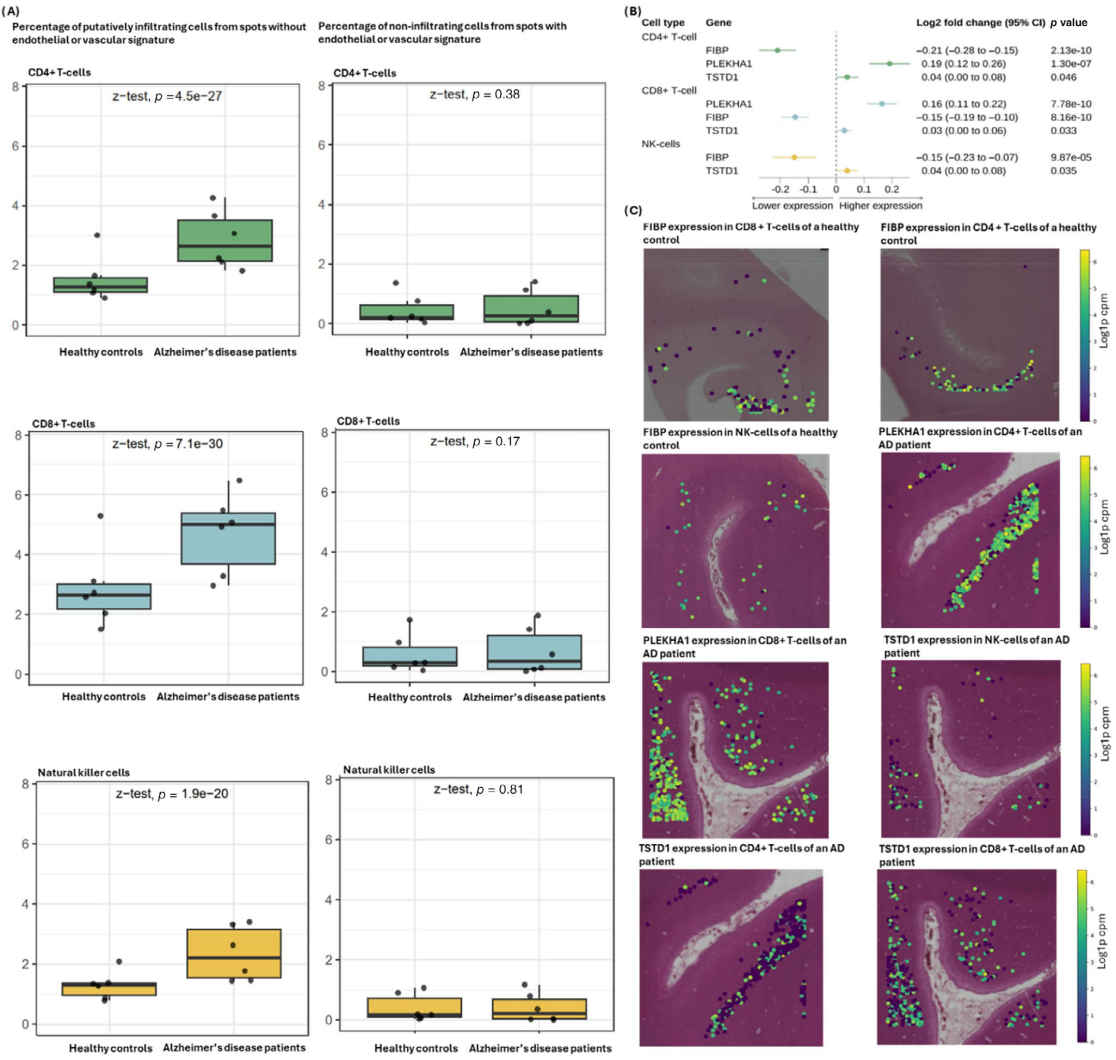
Differential expressions of RNAs in brain‐infiltrating peripheral immune cells between Alzheimer's disease (AD) patients and healthy controls that have consistent effect direction with results from Mendelian randomization and colocalization analyses. (A) Box plots displaying percentage of putatively infiltrating and non‐infiltrating cells in spots with and without endothelial or vascular signatures. (B) Forrest plots showing effect size and direction. (C) Brain tissue samples showing RNA expression patterns that increase AD risk in patients and those that reduce risk in healthy controls.

We examined whether these 13 AD risk‐associated genes were differentially expressed in the corresponding putatively brain‐infiltrating PBMCs between AD patients and heathy controls (Figure 4). Differential expression was detected for three genes: *FIBP*, *PLEKHA1*, and *TSTD1*. The following sections describe the expression patterns of the 13 genes across immune cell types.

### 3.1 CD4^+^ T cells

Among the 10 genes associated with AD risk in CD4^+^ T cells, the associations were generally stronger in the unstimulated in vivo data compared with the stimulated in vitro data (Figure 2A,B and Figures S1A, S2, and 3A). The strongest AD risk‐increasing associations were observed for *BIN1* and *PLEKHA1* in unstimulated cells, with odds ratios (ORs) ranging from 1.95 to 2.80, followed by *TSTD1* and *CTSW* (OR: 1.21 to 1.33). *SCIMP* also showed a considerable risk‐increasing effect, although its high ORs likely reflect model instability due to limited data availability. In contrast, *CTSH* and *HLA‐DQA1* were associated with a reduced AD risk, with ORs between 0.01 and 0.08.

*BIN1*, *CTSW*, and *TSTD1* were associated with an increased AD risk across stimulation time windows from 16 h to 5 days and at different stages of CD4^+^ T‐cell development, whereas *PLEKHA1* elevated risk only 3 h after pathogen stimulation. In stimulated CD4^+^ T cells, *FIBP* was associated with reduced AD risk in both memory and naïve subsets at later stages of the immune response (40 h and 5 days), while *STX4* decreased risk at 16 h and *KAT8* at 5 days after stimulation.

Next, we examined whether the 10 AD risk‐associated genes were differentially expressed in putatively brain‐infiltrating CD4^+^ T cells of AD patients compared with healthy controls (Figure 4 and Table S2). CD4^+^ T cells were enriched in AD brains and localized mainly to the brain parenchyma rather than areas with endothelial or vascular expression profiles. Three genes had directionally consistent associations with the MR findings; *TSTD1* and *PLEKHA1* had higher expression and *FIBP* lower expression in the AD brains.

We then performed pathway enrichment analyses to determine which pathways were overrepresented among CD4^+^ T cell risk genes (Figure S4). In unstimulated CD4^+^ T cells, we identified increased immune response and HLA II receptor assembly and antigen processing. In stimulated CD4^+^ memory T cells, the risk genes enriched in processes affecting glutamanergic synapse, somatodendritic compartment, and dendritic tree that are all key processes in the pathogenesis of AD. For other CD4^+^ T‐cell types, no enrichment was observed, potentially due to a limited number of risk genes.

### 3.2 CD8^+^ T cells

In total, six genes expressed in CD8^+^ T cells were associated with AD risk (Figure 3 and Figures S1B and S3A). *BIN1*, *CTSW*, and *TSTD1* increased AD risk across all CD8^+^ T‐cell subsets, with ORs ranging from 1.06 to 2.92, whereas *FIBP* and *HLA‐DQA1* decreased risk in a subset of cells (OR: 0.04 to 0.58). Following pathogen stimulation, only *CTSW* and *PLEKHA1* were associated with increased risk.

AD brains also showed enrichment of CD8^+^ T cells, which localized predominantly to the brain parenchyma rather than to regions with endothelial or vascular expression signatures. In these putatively brain‐infiltrating CD8^+^ T cells, three genes showed directionally consistent associations with the MR findings (Figure 4). In AD brain samples, *TSTD1* and *PLEKHA1* were expressed at higher levels and *FIBP* at lower levels compared with healthy controls. In addition, when both CD4^+^ T cells and CD8^+^ T cells were analyzed as a single T‐cell group, *CTSW* also showed higher expression in AD brain samples (Table S2).

Pathway analyses indicated that the risk‐associated genes in unstimulated CD8^+^ cells were enriched for pathways related to cellular activation, adhesion, and antigen processing and in stimulated CD8^+^ cells for lytic processes essential to cytotoxic function after stimulation (Figure S5).

### 3.3 B cells

In total, five genes expressed in B cells were associated with AD (Figure 3 and Figures S1C and S3B). In unstimulated B cells, higher *SCIMP* expression was associate with increased AD risk in immature and naïve subtypes, while *BIN1* and *CTSH* increased risk in memory B cells (OR: 1.33 to 3.33). In contrast, *HLA‐DQA1* expression was associated with reduced AD risk across all B‐cell subtypes (OR: 0.83 to 0.90). Following pathogen stimulation, only *HLA‐DRB1* expression was associated with increased AD risk in B cells (all subtypes combined). Of these risk genes, no directionally consistent expression patterns were detected in putatively brain‐infiltrating B cells (Table S2). Pathway analyses indicated that the risk‐associated genes in unstimulated B cells were enriched for pathways related to antigen processing, endosomal activity, regulation and responsiveness to stimuli, and in stimulated B cells for regulation and higher responsiveness to stimuli (Figure S6).

### 3.4 Natural killer cells

In total, five genes expressed in NK cells were associated with AD (Figure 3 and Figures S1D and S3B). *BIN1*, *CTSW*, and *TSTD1* were associated with increased AD risk, with ORs ranging from 1.08 to 1.67, whereas *FIBP* and *HLA‐DQA1* decreased the risk (OR: 0.41 to 0.57). Following pathogen stimulation, only *CTSW* was associated with increased AD risk.

NK cells were likewise increased in AD brains, with their distribution favoring the brain parenchyma over endothelial or vascular‐associated regions. Of the five risk genes, expression of *TSTD1* was higher and *FIBP* lower in the putatively brain‐infiltrating NK cells of AD patients (Figure 4 and Table S2). Pathway analyses indicated that the risk‐associated genes in unstimulated NK cells were enriched for pathways related to increased vesicle processing indicating higher cytotoxic activity, whereas in stimulated cells, no enrichment was observed, potentially due to a limited number of risk genes (Figure S7).

### 3.5 Monocytes

In total, four genes expressed in monocytes cells were associated with AD (Figure 3 and Figure S1E and S3C). *CTSH* increased AD risk in both classical and non‐classical subtypes, with ORs of 1.19 and 1.15, whereas *FCER1G* and *EPHA1‐AS1* decreased the risk (OR: 0.67 to 0.92). Following pathogen stimulation, only *HLA‐DRB1* associated with increased risk. Of these risk genes, no differential expression was detected in putatively brain‐infiltrating monocytes (Table S2). Pathway analyses indicated that the risk‐associated genes in unstimulated monocytes were enriched for pathways related to enhanced responsiveness to immune stimuli and immune receptor activity and after stimulation to higher activity of Golgi, endosome, lysosomal, and vacuolar membranes, as well as plasma membrane complexes involved in processes that form phagosomes in activated monocytes (Figure S8).

### 3.6 Dendritic cells

Four genes expressed in dendritic cells were associated with AD (Figure 3 and Figure S1E and S3C). In unstimulated dendritic cells, *CTSH* increased (OR: 1.17) and *FCER1G* decreased (OR: 0.91) AD risk. Following pathogen stimulation, *HLA‐DRB1* and *HLA‐DQA1* were associated with increased risk. Of these risk genes, no differential expression was detected in putatively brain‐infiltrating dendritic cells (Table S2). Pathway analyses of unstimulated dendritic cells indicated no enrichment, potentially due to a limited number of risk genes, whereas enrichment to positive regulation of immune response and cell adhesion was enhanced in stimulated cells (Figure S9).

### 3.7 Implications for drug targeting

Taken together, among the 13 genes that passed both MR and colocalization analyses, higher expression of *TSTD1* and *PLEKHA1* was associated with increased AD risk, whereas higher *FIBP* expression was associated with reduced risk in both unstimulated and stimulated blood immune cells. These three genes were the only ones that showed differential expression in putatively brain‐infiltrating immune cells of AD patients, in directions consistent with their association with AD risk (Table 1). Differential expression of these genes was also observed across multiple brain‐resident cell types, including astrocytes, microglia, neurons, and oligodendrocytes (Table S2). Among the three genes, *FIBP* showed the strongest differential expression in CD4^+^ T cells; *PLEKHA1* exhibited comparable expression changes across neurons, microglia, and CD4^+^, CD8^+^, and NK cells; and *TSTD1* showed weaker differential expression in CD4^+^, CD8^+^, and NK cells, with magnitudes similar to those observed across resident brain cell types. In addition, *BIN1* and *CTSW* were associated with increased AD risk in both stimulated and unstimulated blood immune cells, whereas all the other identified risk genes were related to AD risk only in either unstimulated or stimulated cells, but not both.

**TABLE 1:** Summary of top Alzheimer's disease‐related genes expressed in immune cells according to Mendelian randomization, colocalization, and spatial single‐cell RNA analyses.

Gene	Association direction in unstimulated PBMCs	Association direction in stimulated PBMCs	Associations between gene expression and AD by PBMC type	Directionally consistent differential expression in brain infiltrating PBMCs in AD patients
*CTSW*	+	+	Naïve and memory CD4^+^ T cells Naïve and memory CD8^+^ T cells NK cells, NK‐recruiting cells	No differential expression
*FIBP*	−	−	Naïve and memory CD4^+^ T cells Memory CD8^+^ T cells NK cells	CD4^+^ T cells CD8^+^ T cells NK cells
*BIN1*	+	+	Memory B cells, Naïve and memory CD4^+^ T cells Naïve and memory CD8^+^ T cells NK cells, NK‐recruiting cells	No differential expression
*TSTD1*	+	+	Naïve and memory CD4^+^ T cells Naïve and memory CD8^+^ T cells NK cells	CD4^+^ T cells CD8^+^ T cells NK cells
*CTSH*	+ for others − for CD4^+^ T cells	No association	Memory B cells Naïve and memory CD4^+^ T cells Dendritic cells Classical and non‐classical monocytes	No differential expression
*PLEKHA1*	+	+	Naïve and memory CD4^+^ T cells Naïve and memory CD8^+^ T cells	CD4^+^ T cells CD8^+^ T cells
*SCIMP*	+	No association	Naïve B cells, Memory CD4^+^ T cells	No differential expression
*FCER1G*	−	No association	Dendritic cells Classical monocytes	No differential expression
EPHA1−AS1	−	No association	Classical and non‐classical monocytes	Expression not observed
*KAT8*	No association	−	Naïve CD4^+^ T cells	No differential expression
*STX4*	No association	−	Naïve and memory CD4^+^ T cells	No differential expression
*HLA‐DRB1*	No association	+	Naïve and memory B cells Dendritic cells Classical and non‐classical monocytes	Expression not observed
*HLA‐DQA1*	−	+	Naïve and memory CD4^+^ T cells Naïve and memory CD8^+^ T cells Naïve and memory B cells Plasma cells NK‐recruiting cells Dendritic cells	Expression not observed

*Note*: Expression of Alzheimer's disease‐related genes in different cell types and at different stages of immune system activation, as well as their differential expression in brain‐infiltrating peripheral immune cells, is shown. A plus sign (+) indicates that high expression of the gene is associated with increased Alzheimer's disease risk, whereas a minus sign (−) indicates a protective association.

Abbreviations: AD, Alzheimer's disease; NK, natural killer; PBMC, peripheral blood mononuclear cells.

Search of the Open Targets database for drugs targeting the proteins coded by the 13 risk genes identified three drugs, all of which were for *HLA‐DRB1*. Two of these, Apolizumab and LYM‐1, are antibody therapies for hematologic cancers currently in Phase II trials. The trial examining the third drug, a small molecule named Plovamer acetate to treat multiple sclerosis, was terminated after Phase II.

## 4 DISCUSSION

Using scRNA GWAS data from unstimulated and stimulated immune cells as well as Mendelian randomization and colocalization analyses, we found that the expression of 13 cell‐ and immune state‐specific genes may have a causal role in the early pathology of AD in healthy individuals. When the immune system was unstimulated, higher peripheral expression of *BIN1*, *CTSW*, *CTSH*, *TSTD1*, *PLEKHA1*, and *SCIMP* genes in B cells, T cells, NK cells, monocytes, and dendritic cells was associated with increased AD risk, whereas elevated expression of *FIBP*, *FCER1G*, *EPHA1‐AS1*, and *HLA‐DQA1* genes was linked to reduced risk. Following stimulation, associations for upregulated *BIN1*, *CTSW*, *TSTD1*, and *PLEKHA1* and downregulated *FIBP* persisted, while new risk‐increasing associations emerged for *HLA‐DRB1* and *HLA‐DQA1* and risk‐decreasing associations for *KAT8* and *STX4*. Spatial transcriptomics analyses identified directionally consistent gene expression changes in putatively brain‐infiltrating immune cells, with *PLEKHA1* and *TSTD1* upregulated and *FIBP* downregulated in NK cells and CD4^+^ and CD8^+^ T cells enriched in AD brain tissue. Pathway enrichment analyses indicated that the cell type‐specific AD risk genes participate in immune system activation, adaptive immunity, cytotoxicity, and structural neuronal pathways. We identified three drugs that have reached Phase II clinical trials targeting *HLA‐DRB1* but none for the proteins encoded by the other risk genes.

Earlier studies showed that higher levels of the anti‐inflammatory monocyte marker CD28^22^ may protect against AD, whereas elevated levels of immune‐modulating CD33,^23^ inflammatory CD38, HLA‐DR,^24^ and IgD,^25^ as well as increased CD20 expression on B cells,^6^, ^26^ are associated with increased AD risk. Extending previous work, our single‐cell analyses provided increased resolution by identifying 13 potential therapeutic targets with specificity to cell type, immune activation state, and disease stage. We further observed that genes associated with AD risk differed partly between unstimulated and stimulated immune cells, suggesting that baseline “tuning” of the peripheral immune system may influence early AD pathogenesis independently of stimulus‐evoked immune responses. Consistent with findings in both unstimulated and stimulated peripheral immune cells, *post mortem* AD brain samples showed lower expression of *FIBP* and higher expression of *PLEKHA1* and *TSTD1* in putatively brain‐infiltrating NK, CD4^+^, and CD8^+^ T cells, suggesting that these genes may represent cell‐specific candidates for early preventive interventions that are independent of immune stimulation, as well as for later therapeutic interventions.

Although the function of *FIBP* is not well characterized, cancer studies have shown that reduced FIBP expression (knock‐out) in human CD8^+^ T cells enhances cytotoxic activity and tumor cell killing.^27^ This observation suggests that higher *FIBP* expression may confer protection against AD by limiting cytotoxic immune activity and thereby attenuating neurodegeneration. The functions of *PLEKHA1* and *TSTD1* in NK and T cells are also not well characterized; however, *PLEKHA1* has been implicated in lymphocyte activation,^28^ suggesting that its increased expression may contribute to aberrant immune activation within the brain.

Of the peripherally significant genes, *CTSW* had the most consistent association with AD. It increased AD risk in all unstimulated and stimulated NK‐cell, CD4^+^ T‐cell, and CD8^+^ T‐cell types and had a higher expression in brain tissue of AD patients when T cells were analyzed as a single group. *CTSW* is a cysteine protease with increasing expression levels with age in the blood.^29^ In autoimmune gastritis and type 1 diabetes, activated CD8^+^ T cells and NK cells infiltrate target tissues and secrete *CTSW* as part of cytotoxic effector functions,^30^, ^31^, ^32^ whereas in breast and endometrial cancers, higher *CTSW* expression has been associated with improved prognosis by slowing tumor growth.^33^, ^34^ Together, these data suggest that *CTSW* might increase AD risk through heightened cytotoxic immune activity.

The strongest peripheral associations (although not as consistent as for *CTSW*) were observed for *BIN1*, with ORs ranging from 1.67 to 3.33. As the second most significant GWAS locus for AD, *BIN1* showed increased AD risk when highly expressed across nearly all unstimulated and stimulated NK cells, CD4^+^ T cells, and CD8^+^ T‐cell subsets, as well as in unstimulated memory B cells. In contrast, spatial analyses showed reduced *BIN1* expression in putatively brain‐infiltrating immune cells from AD patients, suggesting that *BIN1* may primarily act as a peripheral or early marker of immune dysregulation, with a potentially different role at later disease stages or within the brain microenvironment. These findings are consistent with previous MR analyses of plasma *BIN1* protein levels, which also indicated that higher *BIN1* contributed to increased AD risk prior to clinical diagnosis.^6^ In the present study, no association was detected between *BIN1* expression in pathogen‐stimulated B cells and AD risk, which may reflect the absence of B‐cell subtype annotations in the stimulated dataset or limited statistical power. Several cancer studies have linked reduced *BIN1* expression to tumor aggressiveness and worse prognosis, suggesting a pro‐apoptotic role for *BIN1*.^35^

Of the 13 genes identified in our analyses, seven (*BIN1*, *CTSH*, *EPHA1‐AS1*, *HLA‐DQA1*, *KAT8*, *PLEKHA1*, and *SCIMP*) are also among the top hits in AD GWASs.^4^ While these genes have traditionally been studied in neuronal or glial contexts, our findings provide an alternative perspective by suggesting that their effects on AD risk may, at least in part, be mediated through expression in the peripheral immune system, highlighting immune cells as potential mediators of established AD susceptibility loci. Consistent with this interpretation, our observations in putatively brain‐infiltrating peripheral immune cells – together with pathway enrichment results implicating immune activation, antigen processing, neuronal structural pathways, and cytotoxic programs – support the hypothesis that dysregulation of the peripheral immune system may contribute to neuroinflammation through both systemic immune responses and direct central effects of cytotoxic NK and T cells infiltrating the brain. These innate and adaptive immune mechanisms may drive chronic, compartmentalized neuroinflammation, thereby accelerating neurodegeneration and contributing to AD pathology. This pattern parallels pro‐inflammatory and cytotoxic processes observed in severe central nervous system infections and autoimmune diseases such as multiple sclerosis.^36^, ^37^

The strengths of our study include the use of large scRNA GWAS cohorts that describe blood immune cells at different stages of immune activation and in the brain, allowing us to provide detailed data on immune system dysregulation in AD. Our main analyses included MR and colocalization analyses that together can improve discovery of causal risk factors beyond traditional observational methods.^38^ We also obtained convergent evidence from spatial scRNA data locating the immune cells to brain parenchyma, strengthening the validity of our findings.

This study also has important limitations. Although scRNA GWASs provided eQTLs, they did not always have multiple SNPs for each exposure that would have remained after LD pruning. This prevented us from conducting sensitivity MR analyses examining horizonal pleiotropy and outliers. However, we conducted colocalization analyses that validated part of our MR findings. Another limitation is that in vitro stimulation of immune cells may not fully recapitulate the dynamic and co‐stimulatory environment present in vivo. This limitation is reflected in the markedly lower ORs observed in the in vitro‐stimulated compared with in vivo‐unstimulated datasets, suggesting that some relevant associations may have been missed under stimulated conditions. However, the risk genes were partially the same and directionally consistent in unstimulated and stimulated datasets, providing support for the direction of association with in vitro stimulated data. In addition, RNA expression does not always correlate strongly with protein expression, and larger human datasets on single‐cell proteomics are still very limited. Yet some of our findings (for example, those for *BIN1*) are consistent with MR data using plasma proteomics.^6^ Blood‐derived immune cells are rare within brain tissue, and the spatial resolution of current transcriptomic platforms is suboptimal for interrogating such low‐abundance populations. As a result, some of our differential expression analyses were likely underpowered, potentially missing some relevant differentially expressed genes. In addition, the differential expression signals detected in putatively brain‐infiltrating immune cells may partly reflect transcriptional changes in adjacent glial and neuronal populations, in which these three genes were also differentially expressed. Thus, these findings should be interpreted as hypothesis‐generating rather than definitive evidence of immune cell‐specific effects. To detect these immune cells in the brain with greater confidence, we applied the Cell2location^19^ Python package, which uses a Bayesian hierarchical model to integrate single‐cell reference profiles with spatial transcriptomic data. This probabilistic approach provides higher sensitivity for identifying rare cell types compared with other commonly used methods, such as Seurat.

In conclusion, this study identified 13 genes expressed in peripheral immune cells that link early immune system dysregulation in healthy individuals to higher AD risk. Three of these genes were also differentially expressed in putatively brain‐infiltrating NK and T cells in *post mortem* brain tissues from patients with AD, suggesting a potential role in both disease susceptibility and progression. Future experimental studies are needed to define the mechanistic functions of these genes in immune cells and to assess whether they or their protein products represent viable drug targets for AD prevention or treatment.

## AUTHOR CONTRIBUTIONS

Conceptualization: J.V.L., M.S., M.K., R.J.X. Methodology and investigation: J.V.L. and M.S. (overall analytical design, integration, and contextualization of results); J.V.L. (implementation and execution of Mendelian randomization, colocalization, and enrichment analyses); H.‐M.L. (implementation and execution of Mendelian randomization, colocalization, and spatial transcriptomic analyses); N.M., D.G., O.A. (methodological support). Visualization: J.V.L., M.S., H.‐M.L, N.M., M.K. Funding acquisition: J.V.L., R.J.X., M.K. Supervision: M.K., R.J.X. Writing – original draft: J.V.L., M.S. Writing – review and editing: All authors.

## CONFLICT OF INTEREST STATEMENT

The authors declare no conflicts of interest.

## CONSENT STATEMENT

All human subjects who participated in studies used to generate data for this manuscript provided informed consent.

## Supporting information



Supporting information

Supporting information

Supporting information
