# Supplementary material for: Population and single‐cell analyses reveal immune cell‐specific expression profiles associated with Alzheimer's disease risk
Source: Alzheimers Dement. 2026 Mar 22;22(3):e71282. doi: 10.1002/alz.71282 (PMC13093567; doi:10.1002/alz.71282)

Supplementary Figure 1. Mendelian Randomization analyses examining Alzheimer's disease risk associated with genes expressed in peripheral blood mononuclear cells using data from the corresponding expression GWASs and Alzheimer's disease GWASs. Results are shown by immune cell type, including CD4+ T cells (A), CD8+ T-cells (B), B-cells (C), natural killer cells (D), and dendritic cells and monocytes (E). Genes marked with black passed both Mendelian randomization false discovery correction (FDR p-value <0.05) and colocalization analyses (probability of H4 > 0.7) whereas genes marked with grey passed only false discovery correction.

A) CD4+ T-cells

Cell type	Exposure	Number of SNPs	OR (95% CI)	FDR p−value	Colocalization probability
CD4+ effector memory and central memory T cells					
	SCIMP	1	114970.75 (1020.53 to 12952302.87)	<0.001	0.997
	BIN1	1	2.80 (1.95 to 4.02)	<0.001	1.000
	PLEKHA1	1	2.02 (1.45 to 2.82)	0.004	0.708
	TSTD1	1	1.33 (1.16 to 1.53)	0.004	0.895
	CTSW	1	1.21 (1.11 to 1.33)	0.002	0.968
	EML2−AS1	1	0.16 (0.06 to 0.45)	0.033	No overlapping variants
	HLA−DQA2	1	0.01 (0.00 to 0.05)	<0.001	<0.001
CD4+ naïve and central memory T cells					
	GRIN2C	1	912.69 (17.59 to 47365.37)	0.049	0.070
	MSH3	1	9.92 (3.06 to 32.17)	0.014	0.341
	ZNF652	1	7.07 (2.96 to 16.87)	0.002	0.001
	BIN1	1	2.82 (1.96 to 4.06)	<0.001	1.000
	PLEKHA1	1	1.95 (1.42 to 2.68)	0.004	0.726
	STK4	1	1.75 (1.30 to 2.35)	0.018	<0.001
	TSTD1	2	1.33 (1.18 to 1.49)	<0.001	0.895
	CTSW	1	1.22 (1.11 to 1.33)	0.002	0.968
	RP11−138A9.2	1	0.58 (0.44 to 0.77)	0.015	No overlapping variants
	HLA−DRB5	1	0.42 (0.29 to 0.61)	<0.001	0.017
	FAM241A	1	0.31 (0.16 to 0.59)	0.029	No overlapping variants
	SRA1	1	0.23 (0.11 to 0.49)	0.014	0.461
	HLA−DQA2	1	0.12 (0.06 to 0.23)	<0.001	<0.001
	ZYX	1	0.08 (0.04 to 0.18)	<0.001	<0.001
	CTSH	1	0.08 (0.03 to 0.24)	0.001	0.738
	EML2−AS1	1	0.08 (0.02 to 0.32)	0.033	No overlapping variants
	HLA−DQA1	1	0.01 (0.00 to 0.04)	<0.001	0.999

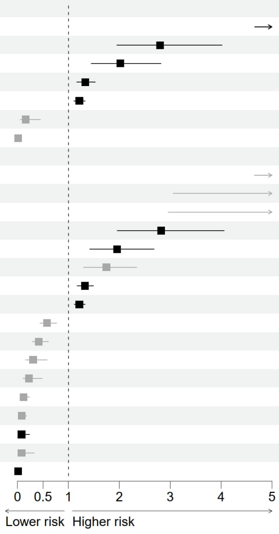

B) CD8+ T-cells

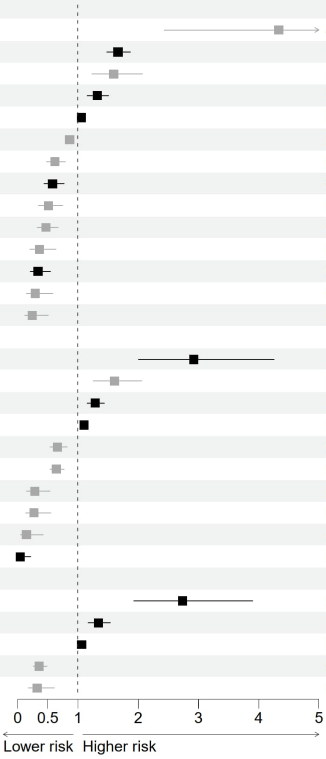

Cell type	Exposure	Number of SNPs	OR (95% CI)	FDR p−value	Colocalization probability
CD8+ effector memory T cells					
	ZNF652	1	4.33 (2.43 to 7.73)	<0.001	0.026
	BIN1	1	1.67 (1.48 to 1.87)	<0.001	1.000
	LAMTOR4	1	1.59 (1.23 to 2.07)	0.033	0.324
	TSTD1	1	1.32 (1.16 to 1.51)	0.005	0.895
	CTSW	1	1.06 (1.03 to 1.09)	0.002	0.968
	HLA−DQA2	2	0.86 (0.82 to 0.90)	<0.001	0.002
	RP11−138A9.2	1	0.62 (0.48 to 0.79)	0.017	No overlapping variants
	HLA−DQA1	1	0.58 (0.43 to 0.77)	0.017	0.997
	WAC−AS1	1	0.51 (0.35 to 0.75)	0.043	0.002
	CEBPZ−AS1	1	0.47 (0.32 to 0.67)	0.005	No overlapping variants
	EML2−AS1	1	0.36 (0.20 to 0.64)	0.033	No overlapping variants
	FIBP	1	0.34 (0.21 to 0.54)	0.002	0.987
	SRA1	1	0.29 (0.14 to 0.58)	0.038	0.451
	SYMPK	1	0.24 (0.12 to 0.51)	0.015	0.024
CD8+ naïve and central memory T					
	BIN1	1	2.92 (2.01 to 4.26)	<0.001	1.000
	STK4	1	1.61 (1.25 to 2.06)	0.017	<0.001
	TSTD1	2	1.29 (1.15 to 1.44)	0.001	0.895
	CTSW	1	1.10 (1.05 to 1.15)	0.002	0.968
	RP11−138A9.2	1	0.66 (0.54 to 0.82)	0.015	No overlapping variants
	HLA−DRB5	1	0.64 (0.53 to 0.77)	<0.001	0.017
	HLA−DQA2	2	0.28 (0.15 to 0.54)	0.013	<0.001
	FAM241A	1	0.27 (0.13 to 0.55)	0.029	No overlapping variants
	EML2−AS1	1	0.15 (0.05 to 0.43)	0.033	No overlapping variants
	HLA−DQA1	1	0.04 (0.01 to 0.22)	0.017	0.998
CD8+ T cells expressing S100B					
	BIN1	1	2.74 (1.92 to 3.90)	<0.001	0.995
	TSTD1	1	1.34 (1.17 to 1.54)	0.004	0.889
	CTSW	1	1.06 (1.03 to 1.09)	0.002	0.968
	HLA−DQA2	1	0.36 (0.26 to 0.49)	<0.001	<0.001
	EML2−AS1	1	0.32 (0.17 to 0.61)	0.033	No overlapping variants

C) B-cells

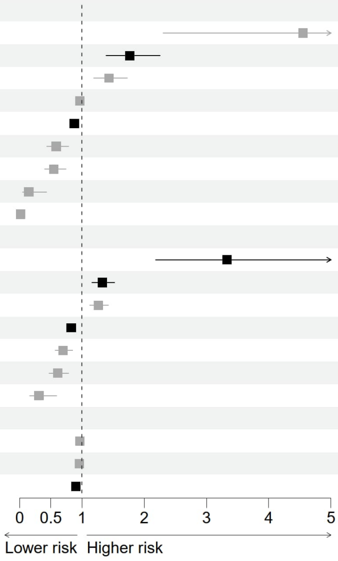

Cell type	Exposure	Number of SNPs	OR (95% CI)	FDR p−value	Colocalization probability
Immature and naïve B cells					
	RP11−960L18.1	1	4.55 (2.30 to 8.99)	0.002	No overlapping variants
	SCIMP	1	1.77 (1.39 to 2.25)	<0.001	0.997
	CTSH	1	1.43 (1.19 to 1.73)	0.015	0.162
	HLA−DQA2	2	0.96 (0.95 to 0.98)	<0.001	0.002
	HLA−DQA1	1	0.88 (0.83 to 0.92)	<0.001	0.797
	PRKD3	1	0.59 (0.44 to 0.79)	0.031	0.306
	HLA−DQB2	1	0.55 (0.40 to 0.74)	0.011	<0.001
	EML2−AS1	1	0.15 (0.05 to 0.43)	0.033	No overlapping variants
	GATS	1	0.02 (0.00 to 0.07)	<0.001	No overlapping variants
Memory B cells					
	BIN1	1	3.33 (2.18 to 5.07)	<0.001	0.998
	CTSH	1	1.33 (1.16 to 1.53)	0.005	0.850
	HLA−DQB1	1	1.27 (1.13 to 1.42)	0.008	0.433
	HLA−DQA1	1	0.83 (0.77 to 0.90)	<0.001	0.932
	FNBP4	1	0.70 (0.57 to 0.85)	0.030	0.015
	HLA−DQB2	1	0.61 (0.48 to 0.79)	0.013	<0.001
	EML2−AS1	1	0.31 (0.16 to 0.60)	0.033	No overlapping variants
Plasma cells					
	HLA−DRB5	1	0.97 (0.95 to 0.98)	0.017	0.017
	HLA−DQA2	1	0.96 (0.94 to 0.97)	<0.001	0.003
	HLA−DQA1	1	0.90 (0.87 to 0.93)	<0.001	0.999

D) Natural killer cells

Cell type	Exposure	Number of SNPs	OR (95% CI)	FDR p−value	Colocalization probability
Natural killer cells					
	BIN1	1	1.67 (1.40 to 2.00)	<0.001	1.000
	STK4	1	1.53 (1.22 to 1.91)	0.018	<0.001
	TSTD1	1	1.26 (1.13 to 1.41)	0.004	0.895
	CTSW	1	1.08 (1.04 to 1.12)	0.002	0.974
	Antisense to HCG27	2	0.87 (0.80 to 0.94)	0.029	No overlapping variants
	HLA−DQA2	2	0.72 (0.65 to 0.80)	<0.001	0.003
	HLA−DRB5	1	0.69 (0.59 to 0.81)	<0.001	0.017
	FIBP	1	0.57 (0.44 to 0.73)	0.002	0.979
	EML2−AS1	1	0.49 (0.33 to 0.73)	0.033	No overlapping variants
	STYX	1	0.30 (0.15 to 0.60)	0.044	0.466
	RP11−308N19.1	1	0.20 (0.08 to 0.46)	0.020	No overlapping variants
Natural killer recruiting cells					
	BIN1	1	1.64 (1.38 to 1.95)	<0.001	1.000
	HLA−DQB1	1	1.33 (1.16 to 1.53)	0.007	0.385
	CTSW	1	1.14 (1.07 to 1.20)	0.003	0.972
	FCER1G	1	0.89 (0.84 to 0.95)	0.036	0.086
	EML2−AS1	1	0.70 (0.57 to 0.85)	0.033	No overlapping variants
	HLA−DQA1	1	0.41 (0.26 to 0.64)	0.008	0.997

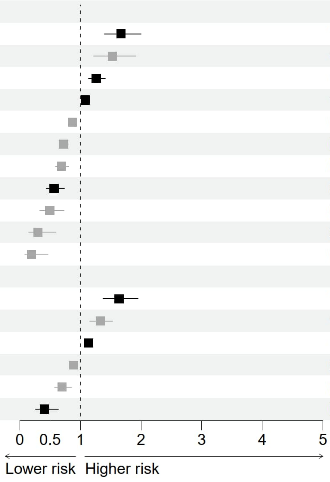

E) Dendritic cells and monocytes

Cell type	Exposure	Number of SNPs	OR (95% CI)	FDR p−value	Colocalization probability
Dendritic cells					
	CTSH	1	1.17 (1.09 to 1.27)	0.006	0.860
	FCER1G	1	0.91 (0.87 to 0.95)	0.001	0.976
Classical monocytes					
	CTSH	1	1.19 (1.09 to 1.30)	0.006	0.861
	HLA−DQA2	2	0.95 (0.93 to 0.97)	<0.001	0.002
	RAC1	1	0.93 (0.89 to 0.97)	0.044	0.045
	FCER1G	1	0.92 (0.89 to 0.95)	0.001	0.976
	WAC−AS1	1	0.71 (0.58 to 0.86)	0.033	<0.001
	EPHA1−AS1	1	0.67 (0.57 to 0.79)	<0.001	1.000
Non−classical monocytes					
	CTSH	1	1.15 (1.08 to 1.22)	0.001	0.861
	LY86	1	1.14 (1.06 to 1.23)	0.044	0.301
	HLA−DQB1	1	1.06 (1.03 to 1.08)	0.003	0.361
	HLA−DQA2	1	0.96 (0.95 to 0.97)	<0.001	0.001
	RAC1	1	0.91 (0.87 to 0.96)	0.044	0.045
	EPHA1−AS1	1	0.72 (0.62 to 0.83)	0.002	0.997
	KCNN4	1	0.71 (0.59 to 0.84)	0.010	0.629

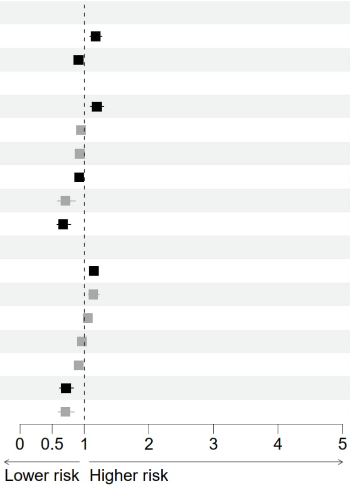

Supplementary Figure 2. Mendelian Randomization analyses examining Alzheimer's disease risk associated with genes expressed in anti-CD3/anti-CD28 human T-activator stimulated CD4+ T-cells using data from the corresponding expression GWASs and Alzheimer's disease GWAS, separately for naïve and specialized CD4+ T-cells (A) and CD4+ memory T-cells (B). Genes marked with black passed both Mendelian randomization false discovery correction and colocalization analyses whereas genes marked with grey passed only false discovery correction.

A) Naïve and specialized CD4+ T-cells

Cell type	Exposure	Number of SNPs	OR (95% CI)	FDR p−value	Colocalization probability
CD4+ naïve T−cells 16 hours					
	GEMIN7	1	1.02 (1.01 to 1.03)	0.001	No overlapping variants
	CTSW	1	1.02 (1.01 to 1.03)	0.001	0.970
	STX4	1	0.99 (0.98 to 0.99)	0.008	0.806
CD4+ naïve T−cells 5 days					
	BIN1	1	1.03 (1.02 to 1.04)	<0.001	1.000
	GEMIN7	1	1.01 (1.01 to 1.02)	0.001	No overlapping variants
	CTSW	1	1.01 (1.01 to 1.02)	0.001	0.971
	TSTD1	1	1.01 (1.01 to 1.02)	0.008	0.895
	KAT8	1	0.98 (0.97 to 0.99)	<0.001	0.996
	FIBP	1	0.97 (0.95 to 0.98)	0.001	0.973
CD4+ naïve T−cells in cycling state 40 hours					
	GEMIN7	1	1.03 (1.01 to 1.04)	0.001	No overlapping variants
CD4+ naïve T−cells in cycling state 5 days					
	CTSW	1	1.01 (1.01 to 1.02)	0.002	0.970
CD4+ naïve T−cells in heat shock protein expressing state 5 days					
	CTSW	1	1.01 (1.01 to 1.02)	0.001	0.971
CD4+ naïve T−cells in interferon expressing state 40 hours					
	CTSW	1	1.01 (1.01 to 1.02)	0.001	0.969
CD4+ T−cells in endoplasmic reticulum stress state 5 days					
	CTSW	1	1.01 (1.01 to 1.02)	0.001	0.971

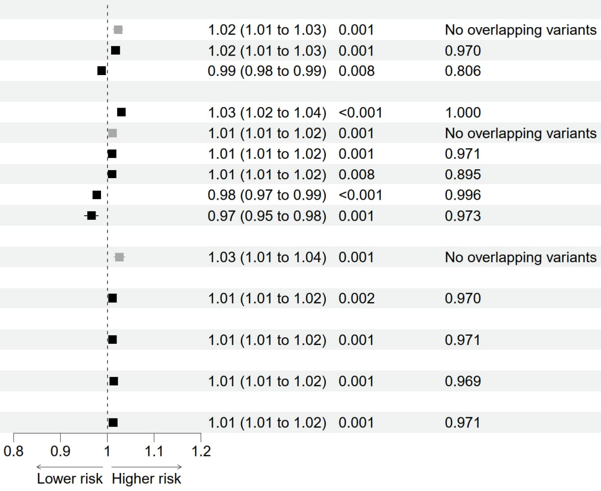

B) CD4+ memory T-cells

Cell type	Exposure	Number of SNPs	OR (95% CI)	FDR p-value	Colocalization probability
CD4+ central memory T-cells 16 hours					
	BIN1	1	1.07 (1.05 to 1.09)	<0.001	0.998
	GEMIN7	1	1.02 (1.01 to 1.02)	0.001	No overlapping variants
CD4+ central memory T-cells 40 hours					
	GEMIN7	1	1.02 (1.01 to 1.03)	0.001	No overlapping variants
	CCNT2-AS1	1	0.98 (0.97 to 0.99)	0.006	0.003
CD4+ central memory T-cells 5 days					
	BIN1	1	1.03 (1.02 to 1.04)	<0.001	1.000
	HSD3B7	1	1.02 (1.01 to 1.02)	0.001	0.014
CD4+ effector memory T-cells 40 hours					
	BIN1	1	1.05 (1.03 to 1.07)	<0.001	0.997
	GEMIN7	1	1.02 (1.01 to 1.03)	0.001	No overlapping variants
	TSTD1	1	1.01 (1.01 to 1.02)	0.008	0.772
	CTSW	1	1.01 (1.01 to 1.02)	0.001	0.970
CD4+ effector memory T-cells 5 days					
	CTSW	1	1.01 (1.01 to 1.01)	0.001	0.971
	TSTD1	1	1.01 (1.00 to 1.01)	0.008	0.895
CD4+ effector memory T-cells re-expressing CD45RA 5 days					
	CTSW	1	1.02 (1.01 to 1.03)	0.001	0.925
CD4+ memory T-cells 16 hours					
	BIN1	1	1.05 (1.04 to 1.06)	<0.001	1.000
	GEMIN7	1	1.02 (1.01 to 1.03)	0.001	No overlapping variants
	CTSW	1	1.02 (1.01 to 1.03)	0.001	0.969
	STX4	1	0.99 (0.98 to 0.99)	0.003	0.763
CD4+ memory T-cells 40 hours					
	BIN1	1	1.07 (1.06 to 1.09)	<0.001	1.000
	TSTD1	1	1.03 (1.02 to 1.05)	0.004	0.874
	GEMIN7	1	1.03 (1.02 to 1.05)	0.001	No overlapping variants
	CTSW	1	1.01 (1.01 to 1.02)	0.001	0.971
	FIBP	1	0.91 (0.88 to 0.95)	0.001	0.936
CD4+ memory T-cells 5 days					
	BIN1	1	1.03 (1.02 to 1.04)	<0.001	1.000
	GEMIN7	1	1.02 (1.01 to 1.02)	0.001	No overlapping variants
	HSD3B7	1	1.01 (1.01 to 1.02)	0.001	0.018
	TMC4	1	1.01 (1.00 to 1.02)	0.037	0.333
	CTSW	1	1.01 (1.01 to 1.01)	0.001	0.971
	CASTOR3P	1	0.98 (0.98 to 0.99)	<0.001	<0.001
	FIBP	1	0.95 (0.93 to 0.97)	0.001	0.981

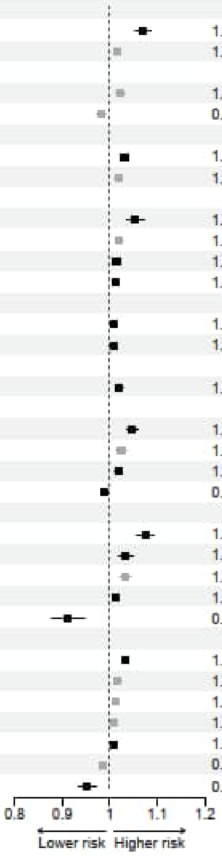

Supplementary Figure 3. Mendelian Randomization analyses examining Alzheimer's disease risk associated with genes expressed in pathogen stimulated peripheral blood mononuclear cells based on the corresponding expression GWASs and Alzheimer's disease GWAS. Results are shown by immune cell type, including CD4+ and CD8+ T-cells (A), B-cells and natural killer cells (B), and dendritic cells and monocytes (C). Genes marked with black passed both Mendelian randomization false discovery correction and colocalization analyses whereas genes marked with grey passed only false discovery correction.

A) CD4+ and CD8+ T-cells

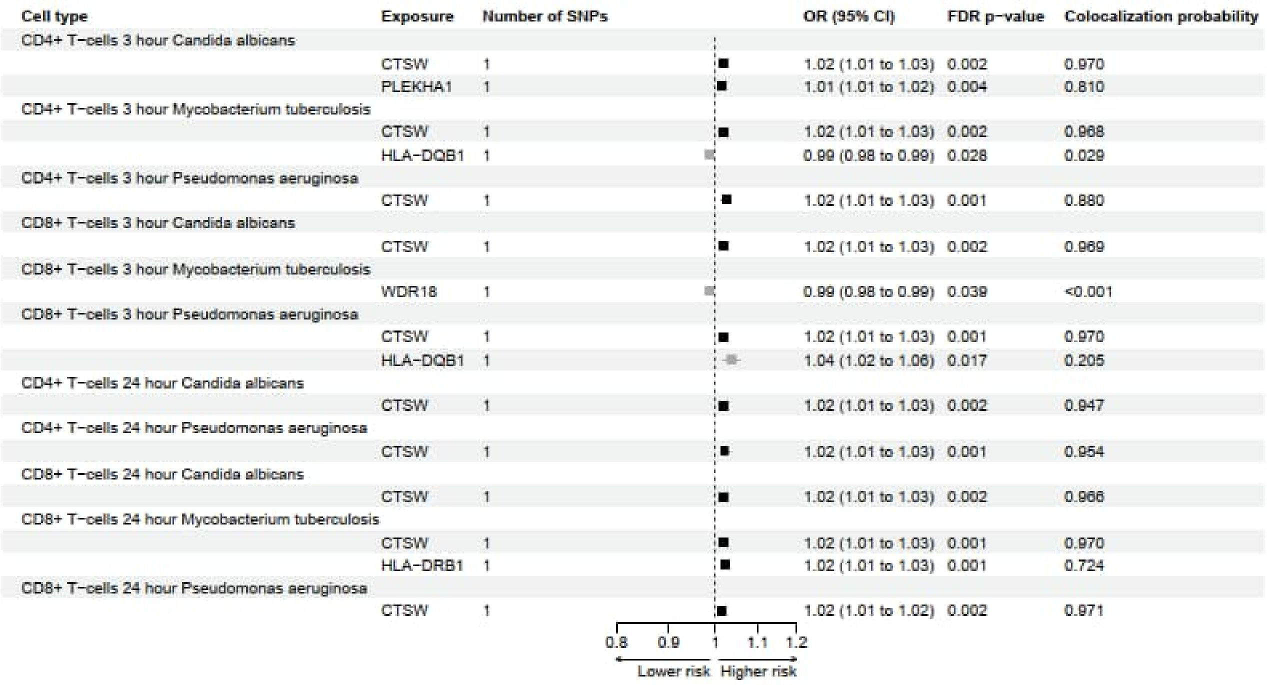

Cell type	Exposure	Number of SNPs	OR (95% CI)	FDR p-value	Colocalization probability
CD4+ T-cells 3 hour Candida albicans					
	CTSW	1	1.02 (1.01 to 1.03)	0.002	0.970
	PLEKHA1	1	1.01 (1.01 to 1.02)	0.004	0.810
CD4+ T-cells 3 hour Mycobacterium tuberculosis					
	CTSW	1	1.02 (1.01 to 1.03)	0.002	0.968
	HLA-DQB1	1	0.99 (0.98 to 0.99)	0.028	0.029
CD4+ T-cells 3 hour Pseudomonas aeruginosa					
	CTSW	1	1.02 (1.01 to 1.03)	0.001	0.880
CD8+ T-cells 3 hour Candida albicans					
	CTSW	1	1.02 (1.01 to 1.03)	0.002	0.969
CD8+ T-cells 3 hour Mycobacterium tuberculosis					
	WDR18	1	0.99 (0.98 to 0.99)	0.039	<0.001
CD8+ T-cells 3 hour Pseudomonas aeruginosa					
	CTSW	1	1.02 (1.01 to 1.03)	0.001	0.970
	HLA-DQB1	1	1.04 (1.02 to 1.06)	0.017	0.205
CD4+ T-cells 24 hour Candida albicans					
	CTSW	1	1.02 (1.01 to 1.03)	0.002	0.947
CD4+ T-cells 24 hour Pseudomonas aeruginosa					
	CTSW	1	1.02 (1.01 to 1.03)	0.001	0.954
CD8+ T-cells 24 hour Candida albicans					
	CTSW	1	1.02 (1.01 to 1.03)	0.002	0.966
CD8+ T-cells 24 hour Mycobacterium tuberculosis					
	CTSW	1	1.02 (1.01 to 1.03)	0.001	0.970
	HLA-DRB1	1	1.02 (1.01 to 1.03)	0.001	0.724
CD8+ T-cells 24 hour Pseudomonas aeruginosa					
	CTSW	1	1.02 (1.01 to 1.02)	0.002	0.971

B) B-cells and natural killer cells

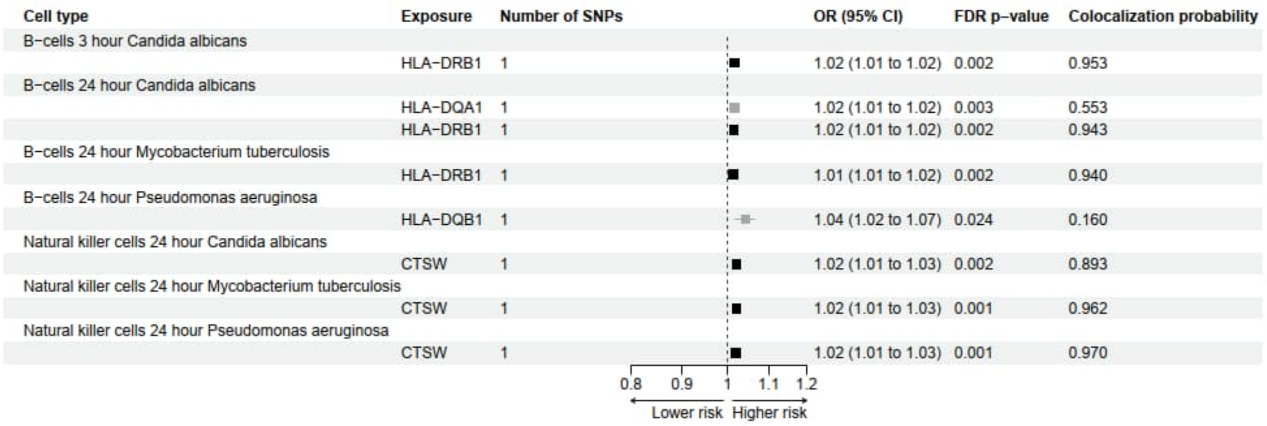

Cell type	Exposure	Number of SNPs	OR (95% CI)	FDR p-value	Colocalization probability
B-cells 3 hour Candida albicans					
	HLA-DRB1	1	1.02 (1.01 to 1.02)	0.002	0.953
B-cells 24 hour Candida albicans					
	HLA-DQA1	1	1.02 (1.01 to 1.02)	0.003	0.553
	HLA-DRB1	1	1.02 (1.01 to 1.02)	0.002	0.943
B-cells 24 hour Mycobacterium tuberculosis					
	HLA-DRB1	1	1.01 (1.01 to 1.02)	0.002	0.940
B-cells 24 hour Pseudomonas aeruginosa					
	HLA-DQB1	1	1.04 (1.02 to 1.07)	0.024	0.160
Natural killer cells 24 hour Candida albicans					
	CTSW	1	1.02 (1.01 to 1.03)	0.002	0.893
Natural killer cells 24 hour Mycobacterium tuberculosis					
	CTSW	1	1.02 (1.01 to 1.03)	0.001	0.962
Natural killer cells 24 hour Pseudomonas aeruginosa					
	CTSW	1	1.02 (1.01 to 1.03)	0.001	0.970

C) Dendritic cells and monocytes

Cell type	Exposure	Number of SNPs	OR (95% CI)	FDR p−value	Colocalization probability
Dendritic cells 3 hour Candida albicans					
	HLA−DQB1	1	1.02 (1.01 to 1.03)	<0.001	0.219
	HLA−DRB1	1	1.01 (1.01 to 1.02)	0.002	0.943
Dendritic cells 3 hour Mycobacterium tuberculosis					
	HLA−DQB1	1	1.01 (1.01 to 1.02)	0.010	0.666
Dendritic cells 3 hour Pseudomonas aeruginosa					
	HLA−DQB1	1	1.01 (1.01 to 1.02)	0.010	0.594
	HLA−DRB1	1	1.01 (1.01 to 1.02)	0.002	0.969
Monocytes 3 hour Candida albicans					
	HLA−DQB1	1	1.01 (1.01 to 1.02)	0.007	0.575
	HLA−DRB1	1	1.02 (1.01 to 1.02)	<0.001	0.950
Monocytes 3 hour Mycobacterium tuberculosis					
	HLA−DQB1	1	1.03 (1.01 to 1.05)	0.039	0.432
Monocytes 3 hour Pseudomonas aeruginosa					
	HLA−DQB1	1	1.01 (1.01 to 1.02)	0.007	0.661
	HLA−DRB1	1	1.02 (1.01 to 1.02)	0.001	0.968
Dendritic cells 24 hour Candida albicans					
	HLA−DQA1	1	1.01 (1.01 to 1.02)	0.007	0.748
Dendritic cells 24 hour Mycobacterium tuberculosis					
	HLA−DQA1	1	1.02 (1.01 to 1.02)	0.009	0.649
Monocytes 24 hour Mycobacterium tuberculosis					
	HLA−DRB1	1	1.05 (1.02 to 1.08)	0.019	0.930

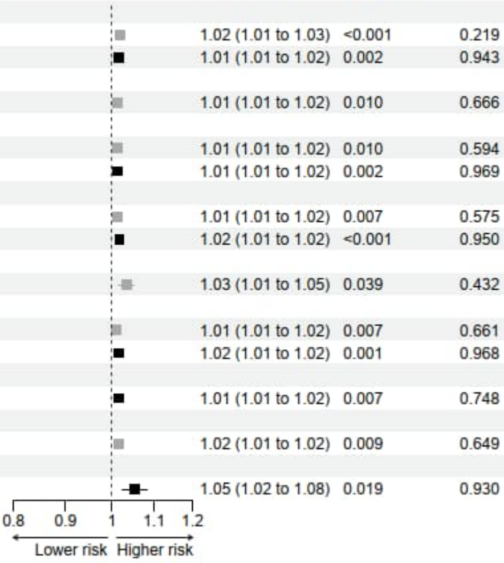

Supplementary Figure 4. Pathway enrichment of false discovery rate correction-significant genes from Mendelian randomization analyses of Alzheimer's disease risk, using RNA expression GWASs of (A) unstimulated CD4+ T-cells and (B) T-activator–stimulated CD4+ T-cells as exposure and Alzheimer's disease GWAS as outcome.

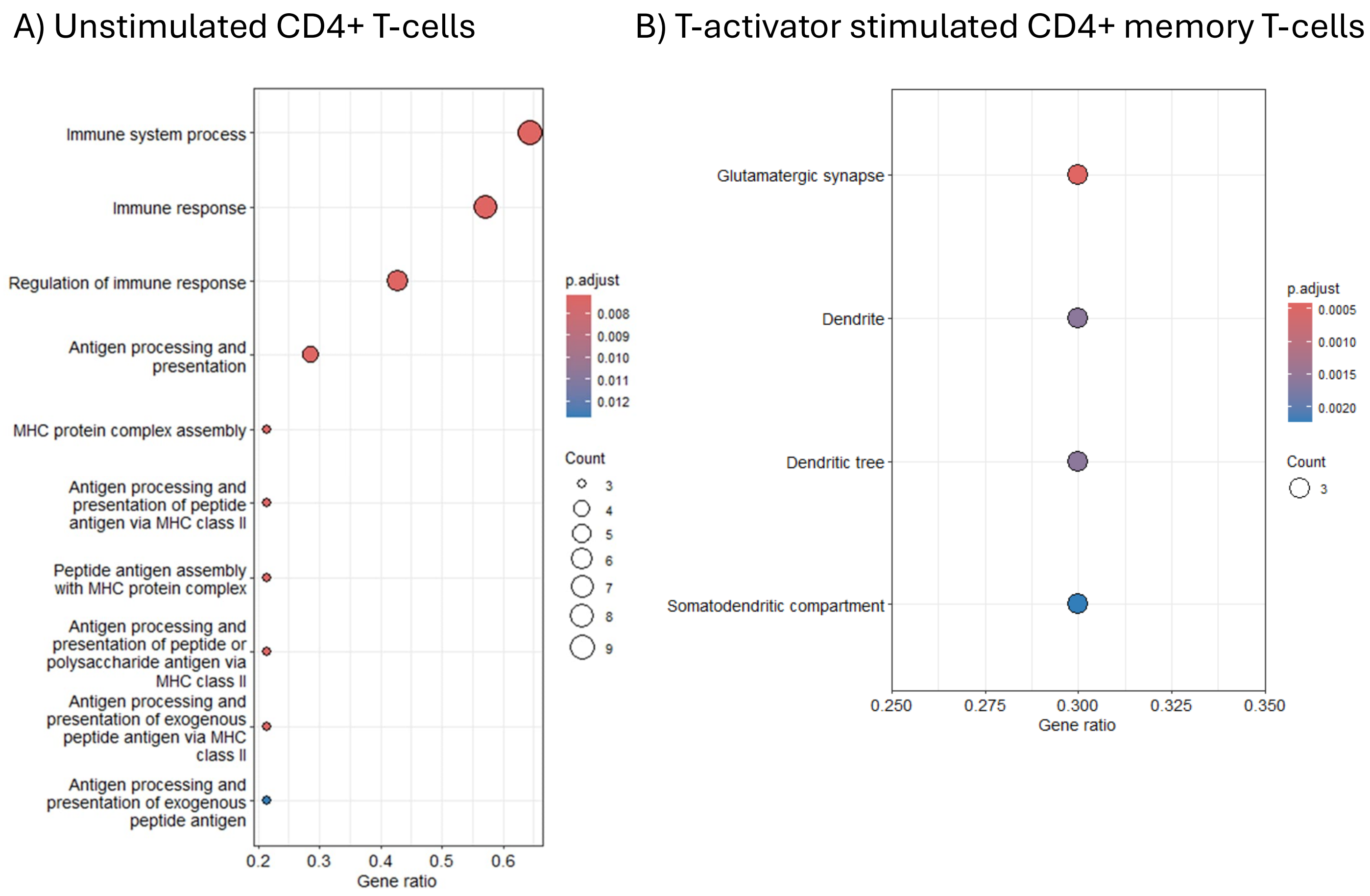

Supplementary Figure 5. Pathway enrichment of false discovery rate correction-significant genes from Mendelian randomization analyses of Alzheimer's disease risk, using RNA expression GWASs of (A) unstimulated CD8+ T-cells and (B) pathogen-stimulated CD8+ T-cells as exposure and Alzheimer's disease GWAS as outcome.

A) Unstimulated CD8+ T-cells

B) Pathogen stimulated CD8+ T-cells

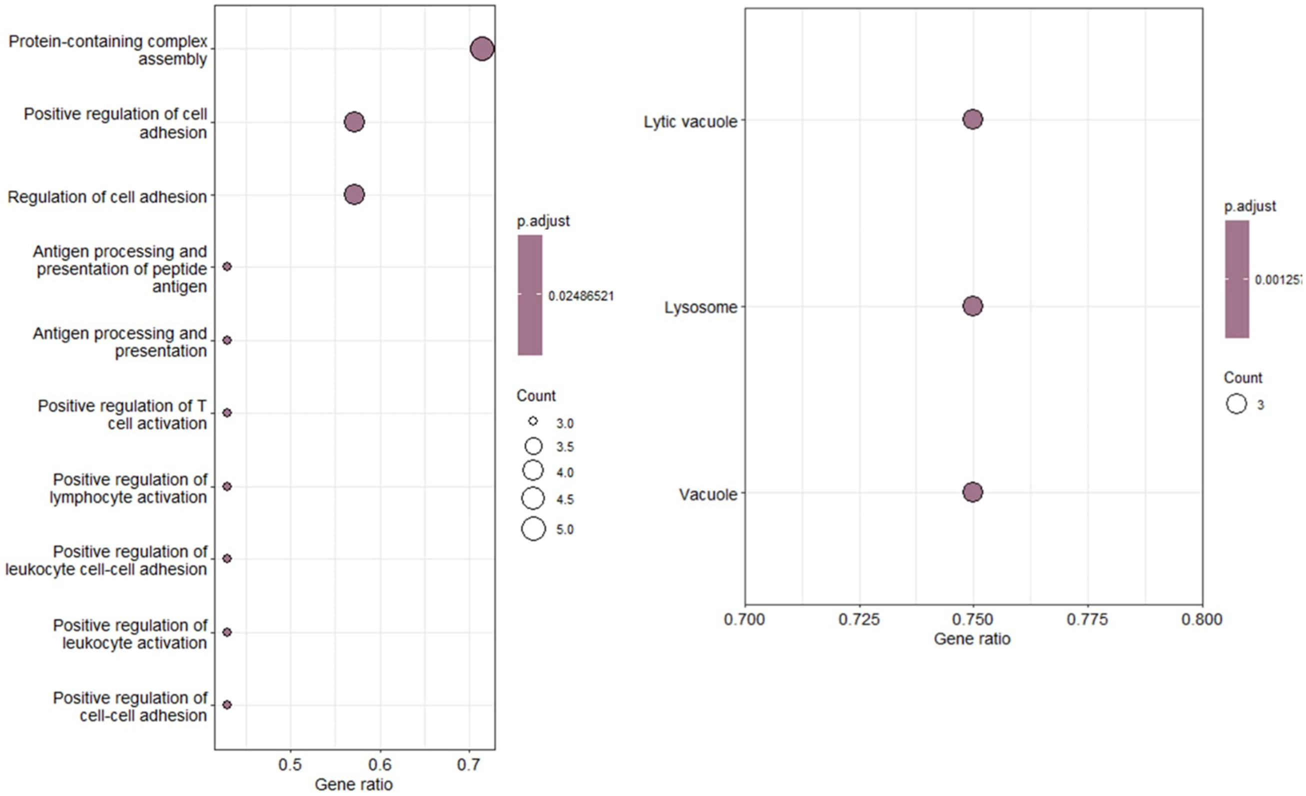

Supplementary Figure 6. Pathway enrichment of false discovery rate correction-significant genes from Mendelian randomization analyses of Alzheimer's disease risk, using RNA expression GWASs of (A) unstimulated immature naïve B-cells, (B) unstimulated memory B-cells, and (C) pathogen-stimulated B-cells as exposure and Alzheimer's disease GWAS as outcome.

A) Unstimulated immature naïve B-cells

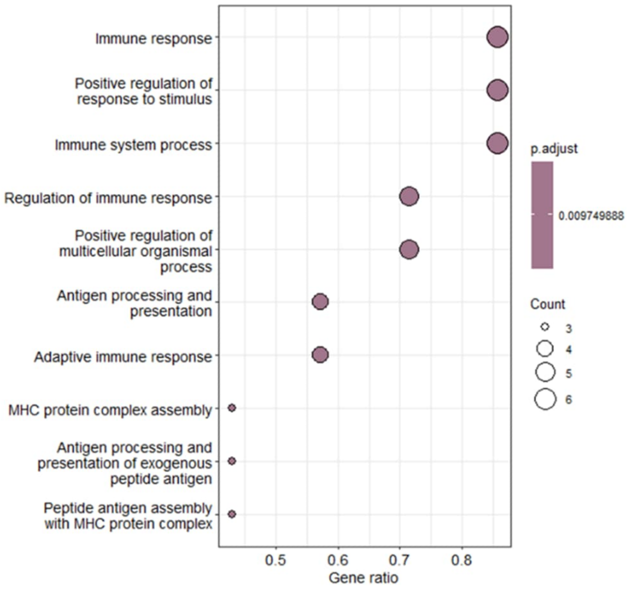

B) Unstimulated memory B-cells

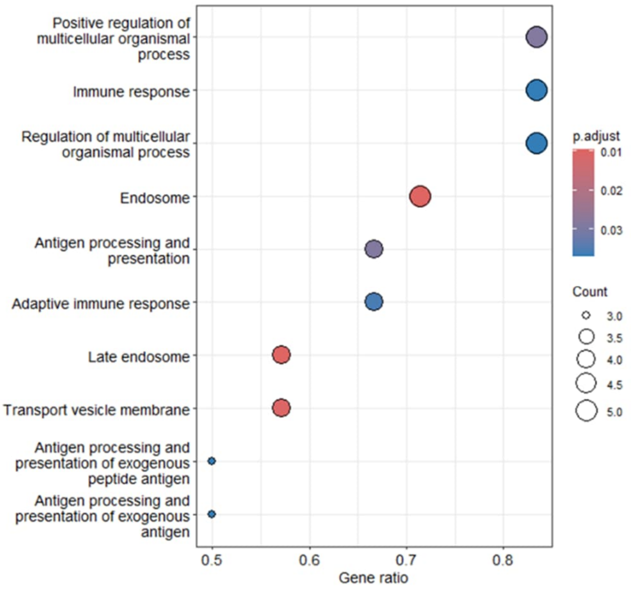

C) Pathogen stimulated B-cells

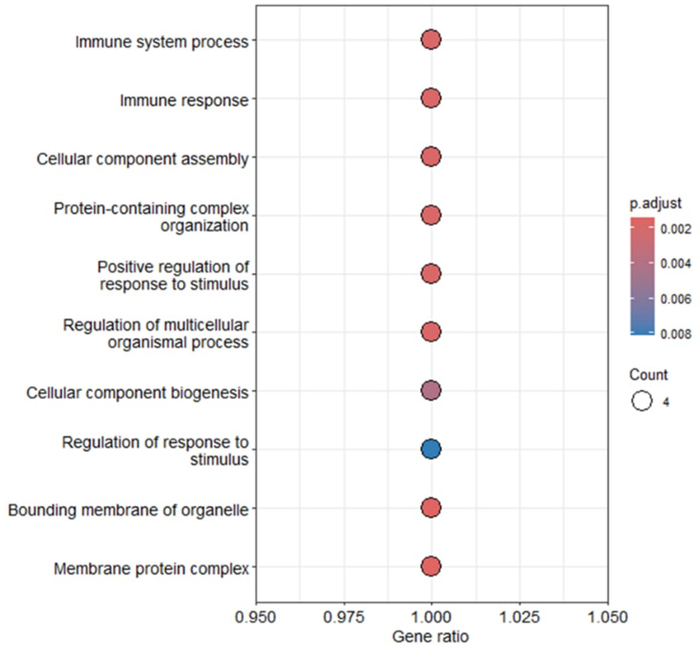

Supplementary Figure 7. Pathway enrichment of false discovery rate correction-significant genes from Mendelian randomization analyses of Alzheimer's disease risk, using RNA expression GWASs of (A) unstimulated natural killer cells and (B) unstimulated natural killer recruiting cells as exposure and Alzheimer's disease GWAS as outcome.

A) Natural Killer cells

B) Natural killer recruiting cells

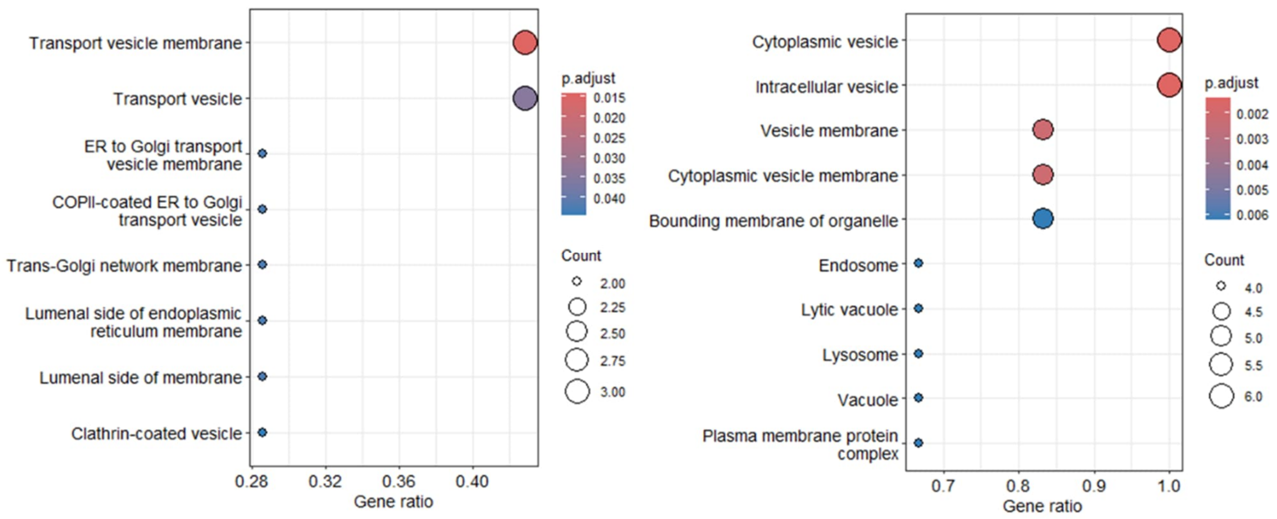

Supplementary Figure 8. Pathway enrichment of false discovery rate correction-significant genes from Mendelian randomization analyses of Alzheimer's disease risk, using RNA expression GWASs of (A) unstimulated classical monocytes, (B) unstimulated non-classical monocytes, and (C) pathogen-stimulated monocytes as exposure and Alzheimer's disease GWAS as outcome.

A) Unstimulated classical monocytes

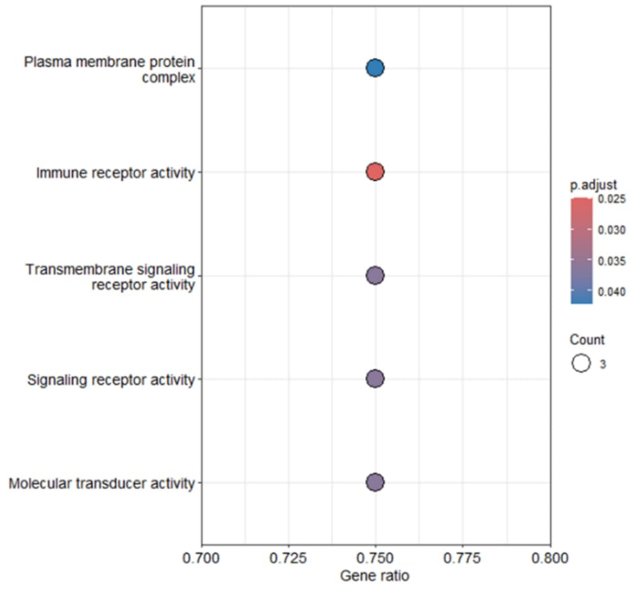

B) Unstimulated non-classical monocytes

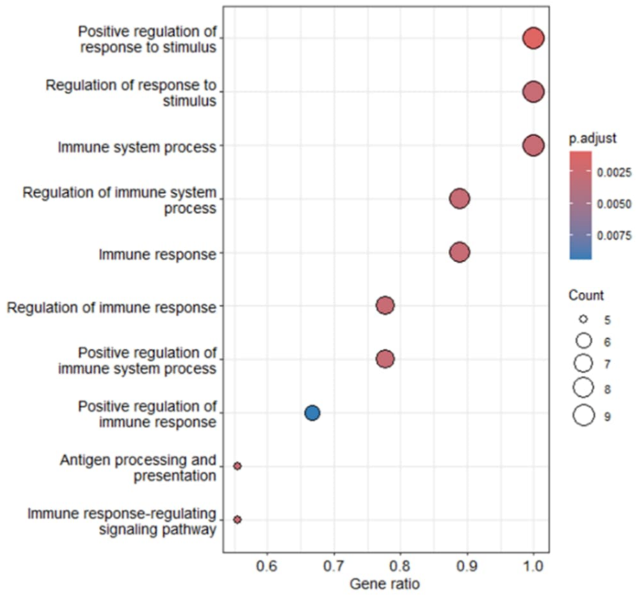

C) Pathogen stimulated monocytes

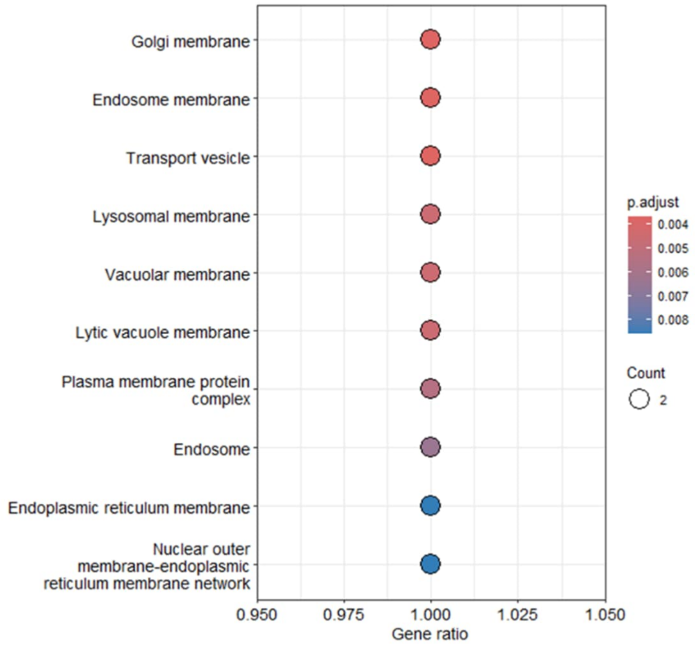

Supplementary Figure 9. Pathway enrichment of false discovery rate correction-significant genes from Mendelian randomization analyses of Alzheimer's disease risk, using RNA expression GWAS of pathogen-stimulated dendritic cells as exposure and Alzheimer's disease GWAS as outcome.

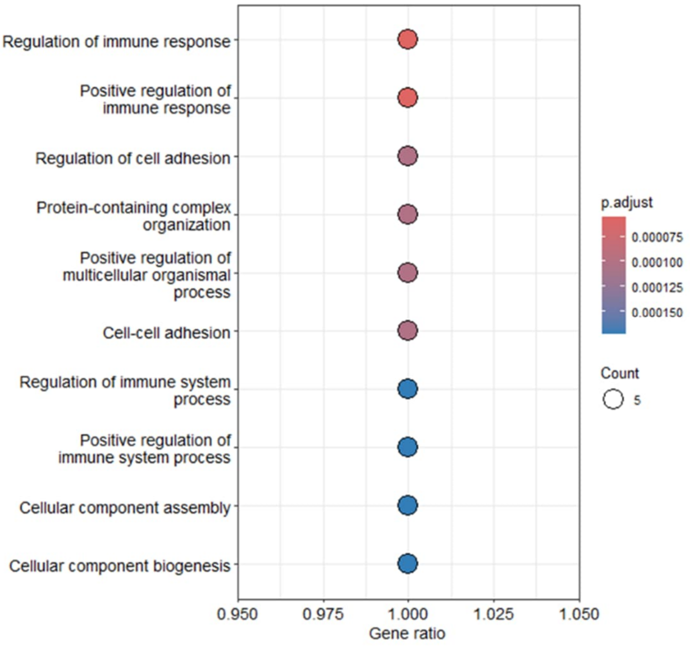

Supplement: Supplementary file 1 — Supporting information [file ALZ-22-e71282-s001.pdf]
